# Phosphorylation and acetylation of mitochondrial transcription factor A promote transcription processivity without compromising initiation or DNA compaction

**DOI:** 10.1016/j.jbc.2022.101815

**Published:** 2022-03-10

**Authors:** Sean D. Reardon, Tatiana V. Mishanina

**Affiliations:** Department of Chemistry and Biochemistry, University of California San Diego, La Jolla, California, USA

**Keywords:** phosphorylation, acetylation, DNA compaction, mitochondrial transcription, AGC, automatic gain control, FA, formic acid, hPKA, human protein kinase A, hPKAc, human PKA catalytic subunit, LSP, light strand promoter, mtDNA, mitochondrial DNA, MTS, mitochondrial targeting sequence, OXPHOS, oxidative phosphorylation, POLRMT, human mitochondrial RNA polymerase, PTM, posttranslational modification, TEV, tobacco etch virus, TFAM, transcription factor A, mitochondrial, TFB2M, transcription factor B2, mitochondrial

## Abstract

Mitochondrial transcription factor A (TFAM) plays important roles in mitochondrial DNA compaction, transcription initiation, and in the regulation of processes like transcription and replication processivity. It is possible that TFAM is locally regulated within the mitochondrial matrix *via* such mechanisms as phosphorylation by protein kinase A and nonenzymatic acetylation by acetyl-CoA. Here, we demonstrate that DNA-bound TFAM is less susceptible to these modifications. We confirmed using EMSAs that phosphorylated or acetylated TFAM compacted circular double-stranded DNA just as well as unmodified TFAM and provide an in-depth analysis of acetylated sites on TFAM. We show that both modifications of TFAM increase the processivity of mitochondrial RNA polymerase during transcription through TFAM-imposed barriers on DNA, but that TFAM bearing either modification retains its full activity in transcription initiation. We conclude that TFAM phosphorylation by protein kinase A and nonenzymatic acetylation by acetyl-CoA are unlikely to occur at the mitochondrial DNA and that modified free TFAM retains its vital functionalities like compaction and transcription initiation while enhancing transcription processivity.

Mitochondria are essential organelles within eukaryotic cells that house the oxidative phosphorylation (OXPHOS) machinery and supply the majority of cellular energy in the form of ATP. As a consequence of their bacterial origin, mitochondria retain copies of a double-stranded (ds), circular DNA genome (mitochondrial DNA, mtDNA), which harbors genes for two mitochondrial-ribosome specific rRNAs, 22 tRNAs, and 13 mRNAs that encode essential OXPHOS-complex proteins (1, 2, 3). The mtDNA is packaged into compact structures called ‘nucleoids’ by mitochondrial transcription factor A (TFAM), a dual-functioning transcription initiation factor and DNA packaging protein, which regulates transcription, replication, and segregation (4, 5, 6, 7, 8, 9, 10, 11). Like most mitochondrial proteins, TFAM is encoded by the nuclear DNA and once transcribed and translated, it is imported into mitochondria, followed by processing into its mature form through removal of its N-terminal mitochondrial targeting sequence (MTS). Once inside the mitochondrial matrix, TFAM binds and bends mtDNA using its two high-mobility group (HMG) box domains, each of which independently bends the DNA 90˚, to introduce an overall U-turn into the DNA. To aid in transcription, TFAM specifically binds the mitochondrial promoter sequences and recruits the mitochondrial RNA polymerase (POLRMT) to the transcription start site (6, 9, 11).

It has been demonstrated both *in vitro* and *in vivo* that TFAM abundance affects transcription initiation and processivity, mtDNA replication, and nucleoid shape in mitochondria, where higher TFAM levels favor transcription and lower levels favor replication (12, 13, 14, 15, 16). It is currently speculated that to quickly respond to changes in cellular energy needs, the mitochondrial nucleoid would need to be fine-tuned locally rather than depend on changing the expression of the nuclear-encoded TFAM. This local remodeling is potentially achieved through posttranslational modifications (PTMs) on TFAM to increase or decrease its stability or affinity for mtDNA, similarly to PTMs of histone proteins in the nucleus and bacterial nucleoid-associated proteins (17, 18). This model is supported by previous *in vivo* and *in vitro* studies. For example, *in vivo* work linked PKA and extracellular signal–regulated protein kinases 1/2 to TFAM phosphorylation, as well as observed acetylation of TFAM isolated from HEK293 cells (19, 20, 21), although detection of modified TFAM in cells required nonphysiological conditions such as overexpression of TFAM and unnatural targeting of PKA to mitochondria. *In vitro* studies of TFAM PTMs and probing of their effects on DNA binding and compaction have often premodified TFAM prior to DNA compaction or utilized PTM mimics in the form of mutations (21, 22). While PTM mimics have been useful to study both acetylation and phosphorylation *in vitro*, studies of proteins other than TFAM show that mimics do not represent the true modification, and therefore, the results must be interpreted with caution. For example, Albough *et al*. showed that lysine mutations to glutamine or arginine (as an acetyl-lysine mimic) resulted in a ∼100-fold *decrease* in enzymatic activity of the yeast histone acetyl transferase compared to its acetylated form, even though this enzyme is normally *activated* by acetylation (23, 24).

In the current work, we fill in the gaps described above to test the PTM model of mitochondrial nucleoid regulation. We find that dsDNA compacted by TFAM leaves TFAM less reactive to both enzymatic phosphorylation and nonenzymatic acetylation than unbound TFAM. This nonreactivity of TFAM should bring into question the current model of local regulation at the mtDNA by TFAM PTMs. We also show that phosphorylated and acetylated TFAM can compact dsDNA with similar efficacy to unmodified TFAM, while quantifying the extent of TFAM acetylation by residue using a proven isotopic labeling approach (25, 26). Lastly, we present that each premodified form of TFAM enhances mitochondrial transcription processivity but leaves transcription initiation unaffected. We conclude that both modified and unmodified TFAM can serve as a compaction protein and transcription initiation factor, but modifications to TFAM may allow modulation of transcription processivity in response to the changing metabolic needs of the cell.

## Results

### Phosphorylation of TFAM is inhibited when bound to circular dsDNA

**Figure 1:**
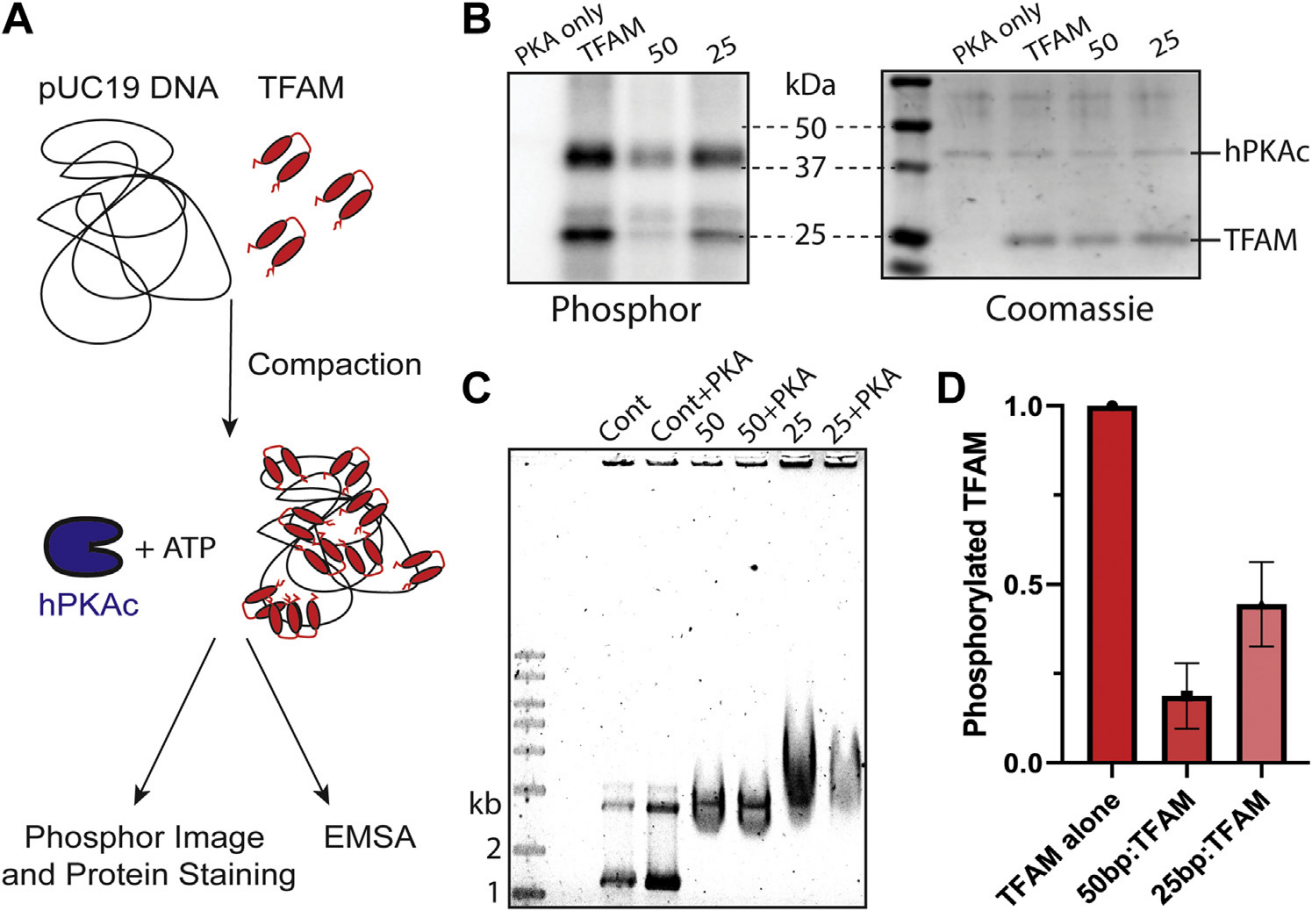
**TFAM is inaccessible to hPKA-catalyzed phosphorylation when bound to DNA.***A*, schematic of the experimental design that includes pUC19 incubation with TFAM (each HMG box in *red*) at varying concentrations and treatment with hPKAc (*blue*) and ATP before analysis with either phosphor imaging and protein staining or an EMSA gel. *B*, a representative phosphor and Coomassie-stained image of the same gel with an hPKAc only sample, a TFAM + hPKAc sample, and compacted samples treated with hPKAc at either 50 bp:TFAM molecule (50) or 25 bp:TFAM molecule (25). *C*, EMSA analysis of TFAM-compacted pUC19 DNA including a pUC19-only control, a control with hPKAc, 50 bp:TFAM molecule (50), 50 treated with hPKAc, 25 bp:TFAM molecule (25), and 25 treated with hPKAc. *D*, quantitative analysis of DNA-bound TFAM phosphorylation at different compaction levels by normalized phosphor signal (n = 3). Each bar represents phosphorylation level as a fraction of the signal from TFAM alone. hPKAc, human PKA catalytic subunit; TFAM, transcription factor A, mitochondrial.

Previous *in vitro* studies of TFAM PTMs and their effect on TFAM’s affinity for DNA either premodified TFAM prior to compacting DNA or relied on mutations mimicking either phosphorylation or acetylation of specific TFAM residues (19, 20, 21). Free TFAM is indeed available for phosphorylation by the catalytic subunit of human PKA (hPKAc) in our hands (lane two in Fig. 1*B*). Premodifying TFAM, however, does not capture the possibility that certain residues may not be accessible to phosphorylation or acetylation when TFAM is involved in DNA compaction, prompting us to check if DNA-bound TFAM is also available for modification. We chose pUC19 DNA because it mimics the circularity of human mtDNA and serves as a template for non-sequence-specific binding and compaction by TFAM. This is in contrast to a previous study that used a short linear 27-base pair (bp) fragment of DNA, which only covers TFAM’s DNA footprint and does not reflect the cooperative nature of circular mtDNA compaction by TFAM (19, 27).

To assess the ability of PKA to phosphorylate DNA-bound TFAM, TFAM was incubated with hPKAc and [γ-^32^P]-ATP either alone or following a 30 min compaction reaction of pUC19 DNA, using either 50 bp DNA:TFAM molecule or 25 bp DNA:TFAM molecule, referred to as “50 bp:TFAM” or “25 bp:TFAM” (Fig. 1*A*). After treatment with ATP and hPKAc, the samples were boiled and run on SDS-PAGE for analysis by phosphor-imaging and subsequently stained for protein content by Coomassie or Krypton fluorescent staining (Fig. 1*B*). The band peak intensities of the phosphor image and protein staining were quantified using ImageQuant software, and each phosphor peak intensity was normalized to its corresponding protein-stained peak intensity (Fig. S1, *A*–*D*). Analysis of three independent experiments shows that hPKAc does *not* phosphorylate DNA-bound TFAM as readily as free TFAM, but that the 25 bp DNA:TFAM sample had more TFAM available for phosphorylation than 50 bp DNA:TFAM (Fig. 1, *B* and *D*). Additionally, an experiment to probe TFAM reactivity across a wider range of DNA compaction levels showed a lower availability of TFAM for hPKA-catalyzed phosphorylation at lower compaction levels (Fig. S2, *A*–*D*). This same experimental setup was performed with unlabeled ATP, and the samples were subjected to a 1% agarose gel for an EMSA to assess DNA compaction, where free DNA migrates faster on the gel than TFAM-compacted DNA (Fig. 1*C*). In agreement with the reduced reactivity of DNA-bound TFAM, the mobility shift of pUC19 DNA by TFAM in an EMSA gel was unchanged by a 2-h incubation with ATP and hPKAc.

### DNA-bound TFAM is less prone to nonenzymatic acetylation

**Figure 2:**
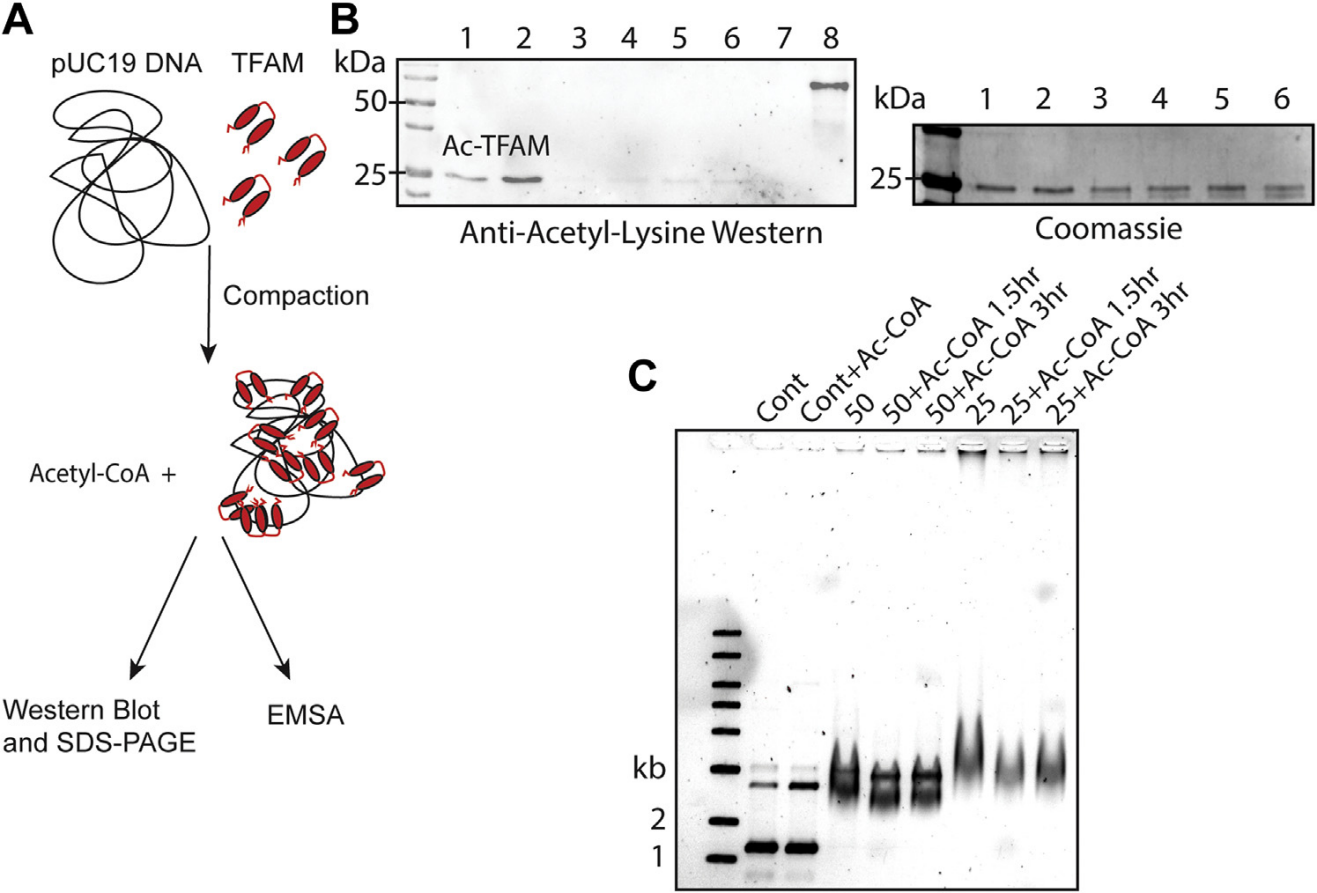
**TFAM is inaccessible to nonenzymatic acetylation when bound to DNA**. *A*, a schematic of the experimental design including compaction of pUC19 DNA by TFAM, treatment of pUC19-bound TFAM with acetyl-CoA and splitting of this sample for analysis by Western blot, SDS-PAGE, and EMSA gels. *B*, a representative Western blot shows the decrease in acetylation of DNA-bound TFAM and the SDS-PAGE representing the loaded proportions of each reaction treated with acetyl-CoA. Lanes 1, TFAM treated with acetyl-CoA for 1.5 h; 2, for 3 h; 3, 50 bp:TFAM molecule treated with acetyl-CoA for 1.5 h; 4, for 3 h; 5, 25 bp:TFAM molecule treated with acetyl-CoA for 1.5 h; 6, for 3 h; 7, TFAM only without treatment; and 8, acetyl-BSA positive control. *C*, EMSA analysis of pUC19 DNA, including a control sample containing pUC19 (Cont), pUC19+acetyl-CoA, 50 bp:TFAM molecule (50), 50 bp:TFAM molecule+acetyl-CoA treated for 1.5 or 3 h, 25 bp:TFAM molecule (25), and 25 bp:TFAM molecule+acetyl-CoA treat for 1.5 or 3 h. BSA, bovine serum albumin; TFAM, transcription factor A, mitochondrial.

The observed decrease in hPKA-catalyzed phosphorylation of DNA-bound TFAM led us to wonder whether nonenzymatic acetylation of TFAM followed similar trends. To test the reactivity of TFAM lysine residues toward nonenzymatic acetylation, in the absence and presence of DNA, pUC19 plasmid DNA was compacted by TFAM before incubation with 5 mM acetyl-CoA at the compaction levels of 50 bp:TFAM and 25 bp:TFAM. After acetyl-CoA incubation, one aliquot of the sample was boiled and subjected to anti-acetyl-lysine Western blot and SDS-PAGE analysis, while another was analyzed by EMSA, to visualize DNA compaction by TFAM (Fig. 2*A*). Western blot analysis of free TFAM, 50 bp:TFAM, and 25 bp:TFAM samples showed extremely reduced TFAM acetylation of compacted samples compared to TFAM alone, while SDS-PAGE analysis of the samples confirmed the presence of TFAM in the DNA-bound samples (Fig. 2*B*). Three independent experiments of this Western blot and SDS-PAGE analysis showed little to no acetylation in DNA-bound TFAM samples. In line with this lack of TFAM modification when on DNA, EMSA gel analysis demonstrated that acetyl-CoA–treated samples incubated at 50 bp:TFAM and 25 bp:TFAM did not show a significant change in mobility compared to the control compacted samples (Fig. 2*C*).

### Acetyl-TFAM and phospho-TFAM are as proficient at compacting circular DNA as unmodified TFAM

After observing reduced susceptibility to modification of DNA-bound TFAM, we wondered if the modification of free TFAM would alter its ability to compact DNA. To answer this question, we measured the compaction ability of premodified TFAM and characterized the extent of each modification to ensure the observed results are not due to remaining unmodified TFAM (*i.e.*, from incomplete phosphorylation or acetylation) — an important detail that was not accounted for in previous studies (19, 22). To this end, TFAM was incubated with ATP and hPKAc or acetyl-CoA, to create working stocks of phospho-TFAM and acetyl-TFAM, respectively. Each stock was subjected to a standard LC-MS/MS workflow that included trypsin digestion and data analysis in MaxQuant. These data reveal that the acetyl-TFAM stock was acetylated at multiple lysine residues, whereas the phospho-TFAM stock carried prominent phosphorylation on S61 only. A previous study identified additional phosphorylation sites on TFAM, for example, S55 and S56 (19). The different result could be due to murine PKA used to phosphorylate TFAM *in vitro* in this prior study *versus* human PKA used in our work. Phospho-S55/S56 TFAM was also detected in human cells in the same study, with an increase in overexpressed TFAM phosphorylation at S55 noted when Lon protease was inhibited, as well as a notable decrease in S55 phosphorylation when hPKA was inhibited with KT5720 and H89. Both KT5720 and H89, however, inhibit many other kinases to the same degree as hPKA, including mitogen-activated protein kinase and phosphoinositide-dependent kinase 1 (28). Considering the inhibition of kinases other than hPKA by these drugs in a human cell, it is unclear whether TFAM was phosphorylated at S55/S56 specifically by hPKA. Additionally, an in-depth study to identify PKA consensus motifs in human cells found that an arginine in the +2 position was the most important factor for substrate recognition by hPKA. The most common consensus sequences were XRXS(T)X > RXXS(T)X > RRXS(T)X (X being any amino acid), whereas less common motifs included KRXS(T)X, RKXS(T)X, and RQXS(T)X (29). S55 and S56 do not fall under any of these motifs with the sequence KPVSSYL, whereas S61 is considered a *bona fide* hPKA target bearing the sequence LRFSKE. Finally, the fact that we do not observe phospho-S55/S56 in our *in vitro* assay with purified components and the presence of a peptide that covers S55 and S56 within our phosphorylated stock’s LC-MS/MS data (Fig. S3) suggests that hPKA does not phosphorylate TFAM at these sites.

As an estimate of the extent of phosphorylation in the phospho-TFAM stock, we used MaxQuant “Mod intensity/Base” value. This value is defined as the sum of all intensities for peptides bearing a modification on a specific residue divided by all of the intensities of the same peptides without the modification. In our case, the S61 phosphorylation “Mod intensity/Base” was 0.99 and thus served as a conservative estimate that our stock is 50% phosphorylated at this residue.

**Figure 3:**
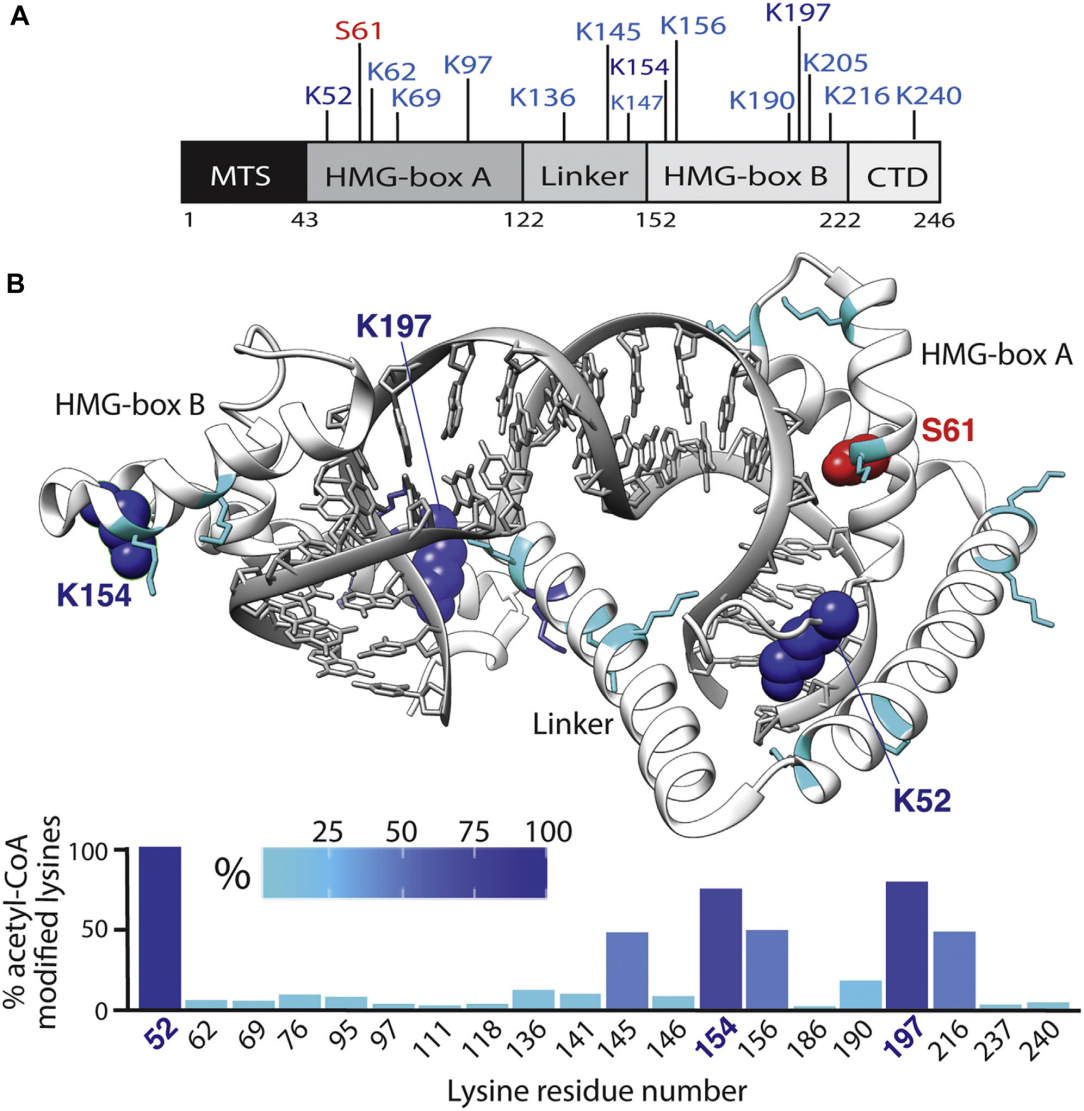
**DNA-free TFAM phosphorylation and acetylation sites following treatment with hPKAc and ATP or acetyl-CoA**. *A*, a schematic showing TFAM’s domains and prominent sites of acetylation (*blue*) or phosphorylation (*red*) identified in this work. *B*, a crystal structure (4NOD) representing DNA-bound TFAM for reference (*white*) with its acetylation sites (*blue*) and phosphorylation site (*red*) indicated. Heavily acetylated lysines are shown in a space-filling model. A *bar graph* represents the relative stoichiometry of lysine acetylation following heavy labeling of unmodified residues with D6-acetic anhydride and LC-MS/MS analysis. The *bar graph* and structure acetylation sites correspond by color in that *cyan* represents lower acetylation and *dark blue* represents higher acetylation. CTD, C-terminal domain; hPKAc, human PKA catalytic subunit; MTS, mitochondrial targeting sequence; TFAM, transcription factor A, mitochondrial.

In order to detect acetylation of TFAM, a typical “control” LC-MS/MS experiment on the acetyl-TFAM stock was performed using only a trypsin digest and typical workflows (see Fig. S3 for MS/MS spectra of acetylated peptides). A second LC-MS/MS experiment was then carried out on the acetyl-TFAM stock as a means to quantify the lysine acetylation stoichiometry by residue using a previously described isotopic chemical acetylation approach (25). Briefly, this protocol uses deuterium-labeled acetic anhydride as an acetylating agent within the LC-MS/MS workflow in which unmodified lysine residues are chemically acetylated following TFAM denaturation and cysteine alkylation. In this experiment, if the lysine is already completely acetylated in the stock TFAM, no “heavy”, or deuterium-labeled, acetylation will be observed, whereas lysines that are only partially acetylated in the stock TFAM will be acetylated partly with “light”, or protium-labeled, acetyl groups present in the stock and partly with “heavy” acetyl groups (added in the acetic anhydride treatment step). The anhydride acetylated sample was sequentially digested with Glu-C and trypsin proteases to ensure small enough peptides are generated for analysis. Upon LC-MS/MS analysis, the peaks identifying an acetyl-CoA, “light” acetylated site will have an analogous “heavy” counterpart in which the intensities can be compared for each acetylated lysine residue (Figs. S5 and S6). Data were processed in MaxQuant using the Andromeda search engine and a custom “heavy acetyl” modification with a mass of 45 Da. We analyzed these data from the aggregate MS1 peptide intensities of peptides bearing only one acetylation site and found that K52 was lacking a heavy-acetyl counterpart altogether indicating it was already completely acetylated in the acetyl-TFAM stock. Additionally, lysines 154, 156, 197, and 216 of the HMG box B and lysine 145 of the linker domain were also highly acetylated within the acetyl-TFAM stock (Fig. 3, *A* and *B*).

We found that the phosphorylated form of TFAM was difficult to work with because it would precipitate when subjected to either size exclusion chromatography or rapid concentration. For this reason, phospho-TFAM was used without further purification other than buffer exchange to remove unreacted ATP. To control for the trace amount of hPKAc used in each downstream compaction or transcription reaction, each of the following set of assays was accompanied by an “hPKAc control”: hPKAc added to a compaction reaction with unmodified TFAM, hPKAc added to a “no-TFAM” control in promoter-independent transcription assays, and hPKAc added to an “unmodified” control for promoter-dependent transcription.

**Figure 4:**
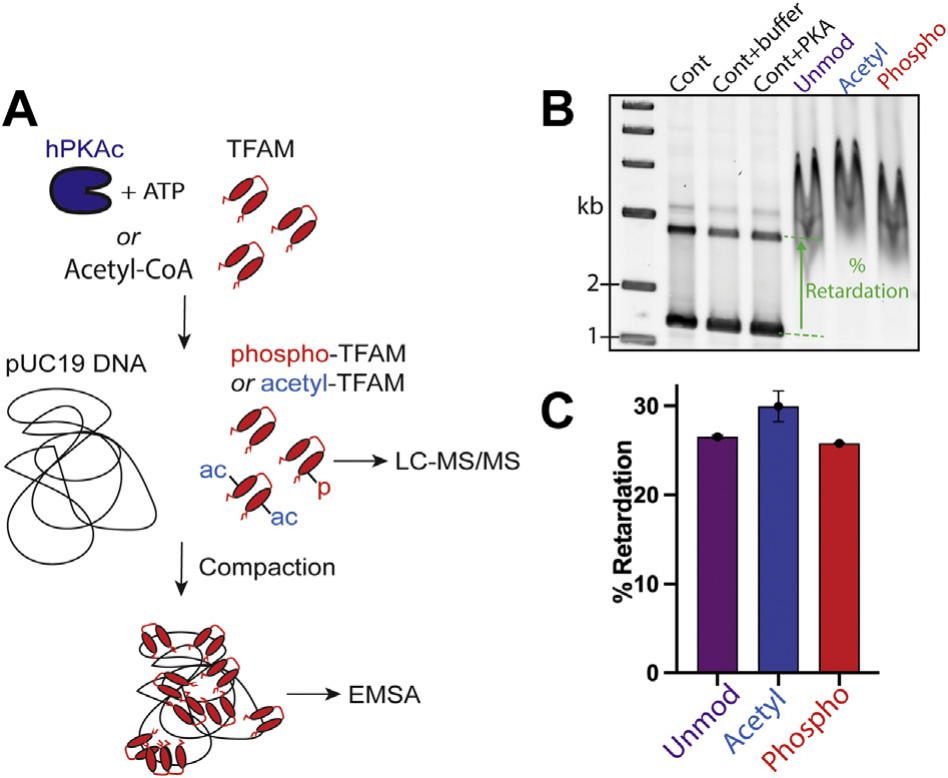
**pUC19 DNA is compacted to a similar extent by unmodified TFAM, acetyl-TFAM, and phospho-TFAM.***A*, schematic of the experimental flow, where TFAM is treated with either hPKAc and ATP or acetyl-CoA prior to DNA compaction. The extent of TFAM modification is quantified by LC-MS/MS, whereas the extent of DNA compaction is assessed by EMSA. *B*, EMSA gel analysis shows the mobility of compacted pUC19 DNA by each version of TFAM, at 50 bp:TFAM molecule. *C*, % retardation of TFAM-compacted pUC19 DNA compared to the migration of unbound pUC19 DNA (n = 3). hPKAc, human PKA catalytic subunit; TFAM, transcription factor A, mitochondrial.

Using the premodified acetyl-TFAM and phospho-TFAM stocks, we assessed the impact of each type of modification on TFAM’s ability to compact dsDNA. To do this, we measured the mobility of pUC19 plasmid DNA compacted at a 50 bp:TFAM ratio. Samples were resolved on an EMSA gel and percent retardation of each compacted DNA band was quantified with respect to naked supercoiled pUC19 DNA. To quantify the shift of the diffuse band of TFAM-compacted DNA, ImageQuant’s band assignment was based on the most intense peak in each lane from the center of the lane (Fig. 4*A*). Analysis of the EMSA gel showed similar compaction capability of phospho-TFAM and acetyl-TFAM compared to untreated TFAM, with acetyl-TFAM displaying a slightly higher percent retardation than unmodified TFAM (*i.e.*, tighter DNA compaction), while phospho-TFAM exhibiting only a slight decrease in percent retardation compared to unmodified TFAM, even though each stock was determined to be heavily modified (Fig. 4*B*) (see LC-MS/MS results above).

### Modified TFAM presents a weaker barrier to processive transcription

**Figure 5:**
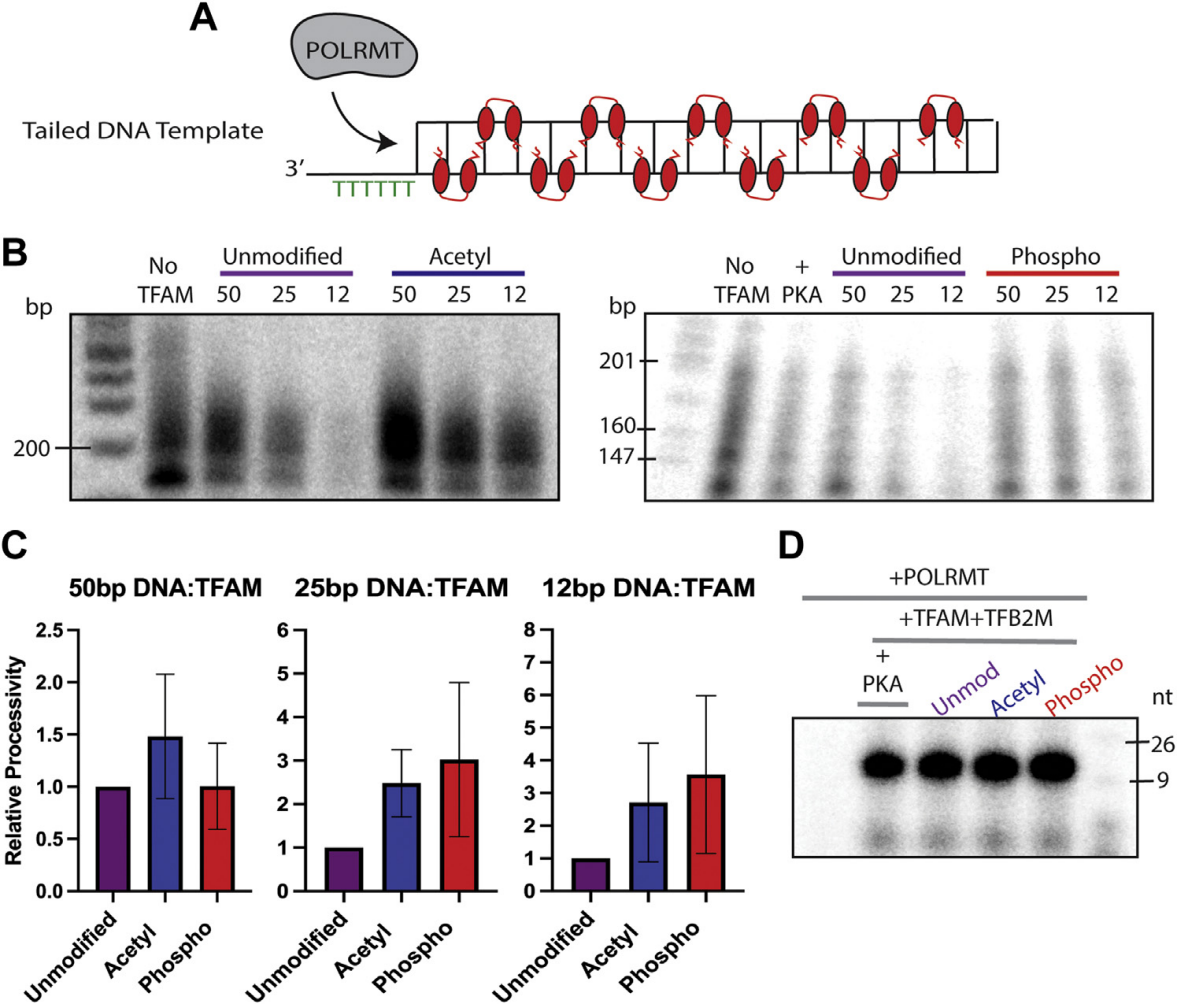
**Phospho-TFAM and acetyl-TFAM allow greater processivity of POLRMT during transcription and aid in transcription initiation to the same extent as unmodified TFAM.***A*, a cartoon depicting the promoter-independent transcription template in which a single-stranded 3′ tail is available for initiation of RNA synthesis by POLRMT alone and the downstream DNA is coated with TFAM “roadblocks”. *B*, representative gels of each promoter-independent transcription assay to compare processivity of POLRMT through unmodified TFAM roadblocks to transcription through acetyl-TFAM and phospho-TFAM roadblocks. *C*, quantified relative processivity of POLRMT through acetyl-TFAM and phospho-TFAM compared to unmodified TFAM at the DNA compaction levels of 50 bp DNA:TFAM, 25 bp DNA:TFAM, and 12 bp DNA:TFAM (n = 3). *D*, a representative gel to assess the initiation of transcription with acetyl-TFAM and phospho-TFAM. nt, RNA product length in nucleotides; POLRMT, human mitochondrial RNA polymerase; TFAM, transcription factor A, mitochondrial.

Having established that TFAM’s ability to compact circular dsDNA is only slightly impacted by acetylation or phosphorylation, we looked to investigate the impact of TFAM modifications on other processes that take place at mtDNA, such as mitochondrial transcription. We chose to determine the processivity of POLRMT during transcription when downstream DNA is compacted by modified forms of TFAM, as a complement to past studies showing that unmodified TFAM acts as a “roadblock” to POLRMT (12). To accomplish this analysis and isolate TFAM’s role as a compaction protein *versus* transcription initiation factor, we used a previously published (12) tailed-transcription template with a free 3′ single-stranded (ss) DNA-tail that allows POLRMT to begin transcription without the help of TFAM and TFB2M (Fig. 5*A*). We observed that POLRMT’s processivity is increased when the downstream DNA is compacted by acetyl-TFAM or phospho-TFAM, compared to unmodified TFAM (Fig. 5, *B* and *C*). To ensure that the decrease in processivity was due to roadblocks by TFAM and not TFAM competing with POLRMT transcription initiation at the ssDNA overhang, we conducted an EMSA analysis of an ssDNA poly-T oligonucleotide and a dsDNA poly-T fragment incubated with TFAM. Like previous studies (19, 30), we found no gel shift of ssDNA incubated with increasing concentration of TFAM, ruling out competition with POLRMT (Fig. S8).

### Modified TFAM assists in transcription initiation at a promoter

We next wondered whether phosphorylation or acetylation of TFAM could play a role in regulating mitochondrial transcription initiation. We used a previously validated transcription template that includes the mitochondrial light strand promoter (LSP) and tested transcription initiation using an NTP mix containing dCTP for termination at the +18 position (11). After reconstitution of the initiation complex, we introduced ATP, UTP, GTP, and dCTP into the reaction and monitored the 18 to 19 nucleotide RNA product formation as an indicator of promoter-initiated transcription efficiency. To this end, we concluded that acetyl-TFAM and phospho-TFAM each initiate transcription to the same extent as unmodified TFAM (Fig. 5*D*).

## Discussion

### TFAM phosphorylation

Here, we assess whether TFAM is accessible for phosphorylation by hPKAc when bound to circular dsDNA. Using these phosphorylation assays, we found that TFAM involved in DNA compaction is less accessible for phosphorylation. Interestingly, we also see that as TFAM concentration is increased, the phosphor-signal increases slightly suggesting some TFAM phosphorylation during the two-hour incubation (Fig. 1*B*). This finding has two possible explanations: (1) there could be “background” phosphorylation of unbound TFAM that is present at very high compaction levels, especially beyond the ratio of 27 bp DNA:TFAM, in which TFAM’s footprint on DNA is occupied or (2) a population of TFAM that is involved in the cooperative nature of compaction predicated upon TFAM–TFAM interactions could be accessible to PKA (21). Given the low level of phosphorylation on DNA-bound TFAM, we propose that TFAM is not normally targeted for modification when part of mitochondrial nucleoids. In support of this proposal, a recent study using a proximity-labeling approach, in which mitochondrially targeted PKA was fused to a biotin ligase (to label PKA’s interacting partners in mitochondria) did not identify TFAM as a “hit”, even though interactions were observed with OXPHOS machinery (31) — interactions repeatedly captured *in vivo* (32, 33).

To the best of our knowledge this work is the first to use phosphorylated TFAM for *in vitro* DNA compaction assays and transcription reactions, as previous studies employed phospho-mimetic TFAM mutations (19). Our EMSA analysis demonstrates that phospho-TFAM compacts pUC19 DNA comparably to unmodified TFAM (Fig. 4). This finding shows that phosphorylated TFAM could presumably still fulfill its function of compacting mtDNA to insulate it from the matrix environment. Furthermore, phospho-TFAM was as capable as unmodified TFAM at assisting POLRMT in initiating transcription (Fig. 5*D*), while on the other hand, presenting a more readily surmountable barrier to POLRMT during transcription *versus* unmodified TFAM. Specifically, our promoter-independent assays indicated that phospho-TFAM is more easily displaced by POLRMT than unmodified TFAM, with a more pronounced difference at higher compaction levels (Fig. 5*B*, right panel). Taken together, our results show that phosphorylation of free TFAM by hPKAc could be used as a way to “loosen” TFAM on the DNA for transcription without sabotaging the ability of TFAM to help initiate transcription or package mtDNA. These findings suggest a more subtle model for regulation of transcription and DNA compaction by TFAM phosphorylation, whereas Suzuki *et al*. proposed degradation of phospho-TFAM by Lon protease as a means to decrease matrix TFAM concentration and thus loosen mtDNA for transcription (19) — a finding that needs to be observed *in vivo* under physiologically relevant conditions, that is, without overexpression of TFAM.

We propose a possible alternative model for TFAM regulation by hPKA. To date, most studies that found PKA residing and active within the mitochondrial matrix have used bovine heart cells; however, whether or not, this is true in other cell types or organisms (*e.g.*, human) is subject to debate (34, 35). A study on both HEK and HeLa cells that utilized fluorescent sensors for PKA activity found that the outer mitochondrial membrane was a privileged location of hPKA activity, whereas the matrix was devoid of significant hPKA activity even though an active soluble adenylate cyclase (which produces cyclic AMP that turns hPKA “on”) was found (36). Based on these previous observations and low accessibility of TFAM to modifications when DNA-bound observed here, we believe it is possible that regulation of TFAM through phosphorylation by hPKAc could be achieved during transport through the outer mitochondrial membrane by hPKAc anchored to mitochondria *via* A-kinase anchoring proteins — a regularly noted phenomenon in various cell types — rather than at the nucleoid itself (37, 38, 39, 40, 41). Future work needs to be done to detect hPKA activity in the mitochondrial matrix across multiple cell types and determine its targets, mechanism for entry, and localization within the matrix.

### TFAM acetylation

Similar to TFAM phosphorylation, we found that nonenzymatic acetylation of TFAM lysine residues by acetyl-CoA was inhibited when TFAM was bound to dsDNA. Baeza *et al*. (25) identified the following trends in nonenzymatic lysine acetylation: (1) increased acetylation if a lysine is surface exposed; (2) increased acetylation if a lysine residue is in spatial proximity to a glutamate or aspartate residue (within 5–7 Å), and (3) reduced acetylation when a lysine is involved in a salt bridge. Given these criteria for acetylation, it is unsurprising to us that TFAM is not reactive when bound to DNA as it is thought that TFAM’s lysine residues play an important role in stabilizing the DNA’s phosphate backbone to allow the bending imparted by TFAM. Our results suggest that TFAM’s role as a compaction protein shields its lysine residues from spontaneous acetylation in the mitochondrial matrix when bound to DNA, but that it is susceptible to acetylation in its free form, perhaps in transit to the nucleoid or through other means that would dissociate it from mtDNA.

In this work, we reproduced experiments done by Fang *et al*. (22) that bound preacetylated TFAM to circular dsDNA, but we also include a full quantitative analysis of our stock’s lysine acetylation stoichiometry from isotopic labeling and LC-MS/MS. In doing so, we further characterized the specific lysine reactivity of TFAM under a long exposure to 5 mM acetyl-CoA. Our results point to K52 as the most reactive residue by far, as a heavy-labeled K52 indicative of an unmodified lysine was not detected during data analysis (Fig. 3*B*). This outcome for K52 agrees well with a similar conclusion by Fang *et al*. based on the analyzed intensities of each modified residue as a measure of modified peptide abundance. However, using isotopic labeling approach, we found that residues K154 and K197 were the next most reactive, whereas Fang *et al*. reported that K69 was the second most reactive lysine residue (found to be only mildly reactive in our analysis, Fig. 3*B*). The reason for this discrepancy lies in the different data analysis approaches: when we performed Fang *et al*.’s analysis of acetyl-TFAM without isotopic labeling using acetylated peptide intensities, we arrived at the same result as Fang *et al*. (Fig. S4). We believe that the isotopic labeling method reports more accurately on lysine reactivities by avoiding peptide bias during LC-MS/MS steps that favor specific residues, such as K69.

We confirmed that heavily acetylated TFAM can still bind and compact circular dsDNA to a slightly higher degree than unmodified TFAM (Fig. 4), in line with the observations by Fang *et al*. (22). Similar to the behavior of phospho-TFAM, we observed that acetyl-TFAM is more easily navigated through by POLRMT during promoter-independent transcription, with a higher impact on processivity with increasing DNA compaction level (Fig. 5*B*). We also note that acetyl-TFAM is equally potent in promoting transcription initiation as unmodified TFAM and phospho-TFAM. Taken together, these data provide a model in which TFAM can be acetylated when unbound to DNA and in this state, it can still promote transcription initiation and compact mtDNA, but serves as a weaker roadblock to the mitochondrial transcription machinery.

## Conclusion

In the present work, the consequences of phosphorylation and acetylation on TFAM’s multiple functions in mitochondria (DNA packaging, transcription initiation, and processivity) are tested. In doing so, we found that many TFAM functionalities are unaffected by the PTMs, but that transcription processivity through the physical barrier created by these modified TFAMs is increased. We suggest that TFAM has likely evolved to operate within the mitochondrial matrix rich in acetyl-CoA as a byproduct of metabolism by bearing many inconsequential acetylated sites that do not disrupt the vital functions of TFAM. We show that TFAM continues to fulfill its role in the compaction of DNA, likely so that mtDNA is continually protected from damage, for example, by the reactive oxygen species in the matrix. Modified TFAM maintains the ability to recruit and aid POLRMT in sequence-specific transcription initiation from the LSP, a function that is particularly important to mtDNA replication as the primer for replication is the transcription termination product from the LSP (42). Most notably, our work supports that TFAM PTMs impart subtle transcription regulation by allowing processivity of POLRMT through the TFAM-coated DNA. Future studies could probe the roles that these modified TFAM forms could play in mitochondrial replication processivity as well as transcription processivity through these “roadblocks” in the presence of transcription elongation factor that clamps POLRMT on DNA. Additionally, TFAM has been missing as a target of the mitochondrial deacetylase, SIRT3, in all studies except one, in which K154 was identified as the only target (43, 44, 45). Determining whether or not other residues on TFAM are regulatable *via* sirtuin-catalyzed deacetylation would help to solidify the acetylated sites as more consequential to TFAM regulation within the mitochondria.

## Experimental procedures

### Purification of TFAM, hPKAc, TFB2M, and POLRMT

Refer to Table S1 for purification buffer components. Constructs encoding TFAM (amino acids 43–246, lacking its N-terminal MTS) with an N-terminal tobacco etch virus (TEV) protease-cleavable hexahistidine (His_6_) tag, and POLRMT (amino acids 43–1230, lacking its N-terminal MTS) were received in the pPROEXHtb vector as generous gifts from the Patel lab (Rutgers University). hPKAc (amino acids 2–351) with a SUMO protease cleavable N-terminal His_6_ tag was gifted as an expressed cell pellet by the Taylor lab (UCSD). TFB2M (amino acids 20–398) fused to a TEV protease-cleavable maltose binding protein-His_6_ tag was provided in a custom pET28 vector as a generous gift from the Garcia-Diaz lab (Stony Brook University). TFAM, POLRMT, and TFB2M were expressed in the BL21(DE3)-RIPL Codon plus *E. coli* strain. Cell pellet containing expressed TFAM was lysed by sonication before running it through a HisTrap column (Cytiva). The eluent was dialyzed overnight and incubated with TEV protease at a 1:30 ratio (TEV:TFAM). The dialyzed sample was then passed through a HisTrap column again to capture the untagged TFAM in the flow-through. The flow-through was then purified *via* a HiTrap Heparin column (Cytiva), and TFAM-containing fractions were collected for concentration and storage. TFB2M was purified following the same steps as TFAM. For purification of POLRMT, the cell lysate treated with polyethyleneimine and precipitation by ammonium sulfate, followed by isolation using a HisTrap column, CaptoDEAE resin (Sigma), and a HiTrap Heparin column. The hPKAc pellet was lysed and purified using Ni-NTA resin. The resin eluant was dialyzed and incubated at 4 °C with SUMO protease at a 1:100 protease to protein ratio. The dialyzed sample was then incubated with Ni-NTA resin, and the sample application was collected for concentration. All proteins were concentrated using either the 3K or 50K MWCO filter (Amicon) and stored at −80 °C prior to use. All buffers used are listed in the Table S1 in the SI.

### Phosphorylation of DNA-bound TFAM and EMSA

TFAM was incubated with pUC19 plasmid DNA in an incubation buffer (50 mM Tris–acetate pH 8.0, 100 mM potassium acetate, 5 mM magnesium acetate, and 1 mM DTT) for 30 min at 37 °C. A mock-incubated sample containing only pUC19 plasmid DNA was incubated in the incubation buffer and loaded as a control. A w/v ratio of 6 ng/μl of plasmid was used in each sample. Each compacted sample was split into a radioactive and nonradioactive phosphorylation reaction that maintained an equal amount of TFAM per reaction. To the radioactive TFAM sample, 10 nM hPKAc and trace [γ-^32^P]-ATP (PerkinElmer) was then added and labeled for 1.5 h before the addition of 0.3 mM unlabeled ATP (HPLC purified, Cytiva) to complete phosphorylation. The reaction was continued for two more hours, boiled for 10 min at 95 °C to quench and denature the proteins, and loaded onto an SDS-PAGE gel for electrophoresis. After electrophoresis, a phosphor screen was exposed overnight to the SDS-PAGE gel and imaged on a Typhoon FLA 9500 (Cytiva). The same gel was then either stained with Coomassie blue or Krypton Stain (ThermoFisher Scientific) and imaged for protein content. The nonradioactive TFAM-compacted DNA samples were treated with 10 nM hPKAc and 0.3 mM ATP immediately after compaction and incubated at 37 °C for 2 h. In addition to these unlabeled phosphorylation reactions, the mock-incubated sample was split into a control-containing DNA, hPKAc, and ATP without TFAM. These samples were then loaded into a 1% agarose EMSA gel with 10% glycerol and run at 100 V at 4 °C for ∼3.5 h in an EMSA running buffer (50 mM Tris–acetate pH 8.0, 2.5 mM EDTA). The EMSA gel was stained with SYBR Gold (ThermoFisher Scientific) for 1 h to visualize the DNA. Band peak intensities were analyzed with ImageQuant software and plotted in PRISM (Fig. S1).

### Acetylation of DNA-bound TFAM, Western blotting, and EMSA

pUC19 plasmid was compacted by TFAM as above and split into acetylation reactions in which mock-incubated pUC19 plasmid (plasmid DNA in the incubation buffer), TFAM alone, 50 bp:TFAM, or 25 bp:TFAM samples were incubated with 5 mM lithium-acetyl-CoA (Sigma) for 1.5 h or 3 h. Each sample was split to maintain an equal amount of TFAM per sample. After incubation, each acetylation reaction was split for Western blot, EMSA, and SDS-PAGE analyses. For SDS-PAGE analysis, a 4 μl aliquot of 50 bp:TFAM and 2 μl aliquots of the TFAM only and 25 bp:TFAM samples (23 ng TFAM) were boiled and run on a 12.5% SDS-PAGE. For EMSA analysis, 9 μl of each reaction (92 ng TFAM) was loaded onto a 1% agarose gel with 10% glycerol and run at 4 °C for ∼3.5 h in the EMSA running buffer listed before. For Western blot analysis, 16 μl of 50 bp:TFAM reaction and 8 μl of each 25 bp:TFAM reaction and TFAM alone (190 ng TFAM) was boiled and run on 12.5% SDS-PAGE. Protein was transferred onto a nitrocellulose membrane (BioRad) using a Turboblot (BioRad). Detection used anti–acetyl-lysine antibody (Cell Signaling Antibody) according to manufacturer’s details and goat-anti-rabbit horseradish peroxidase-linked secondary antibodies. Image analysis was conducted using ImageQuant software (see SI).

### Acetylation and phosphorylation of free TFAM

Acetylation of TFAM stocks was conducted by mixing TFAM and 5 mM lithium-acetyl-CoA (Sigma) into an acetylation buffer (100 mM HEPES–NaOH pH 8.5, 1 mM EDTA) and incubating for 12 h at 37 °C. The reaction was then buffer exchanged using a 3K MWCO filter (Amicon) into the TFAM storage buffer (Table S1) following manufacturer’s instructions. Bulk phosphorylation of TFAM was conducted by diluting stored TFAM aliquots in a phosphorylation buffer (50 mM Tris–acetate pH 8.0, 100 mM potassium acetate, 5 mM magnesium acetate, 10 nM hPKAc, 0.2 mM ATP, 50 nM bovine serum albumin, and 2 mM DTT) and incubated at 30 °C for 16 h. The reaction was then immediately used for DNA compaction and LC-MS/MS analysis. Phospho-TFAM stocks were buffer exchanged into the TFAM storage buffer using an Amicon 3K MWCO filter to remove ATP and stored.

### Sample preparation, chemical acetylation, and double digestion for mass spectrometry

Approximately, 2.5 μg of each TFAM, acetyl-TFAM, and phospho-TFAM was used for LC-MS/MS identification of PTMs, and 1 μg was used in acetyl-lysine labeling studies. For initial PTM analysis phospho-TFAM, acetyl-TFAM, and unmodified TFAM, samples were transferred to 10 K MWCO filter (Millipore) unit that was prefilled with 100 μl 8 M urea and 50 mM ammonium bicarbonate pH 7.4 buffer. While in the filter, proteins were reduced with 5 mM tris(2-carboxyethyl)phosphine at 30 °C for 1 h and alkylated with 15 mM iodoacetamide in the dark at room temperature for 30 min. The buffer was then exchanged to 1 M urea, 50 mM ammonium bicarbonate, and the samples were recovered from the Amicon tube into a microcentrifuge tube. Samples were subjected to overnight digestion with MS-grade Trypsin/Lys-C mix (1:25 enzyme/substrate ratio). Following digestion, the samples were acidified with formic acid (FA), cleaned up with C18 tips, and the extracted peptides were lyophilized to near dryness.

For acetylation with deuterated acetic anhydride, acetyl-TFAM (product of acetyl-CoA treatment) was subjected to double-digestion with Glu-C protease followed by Trypsin/Lys-C mix as described by Baeza *et al*. (25) with some modifications. TFAM samples were transferred to 10 K MWCO filter (Millipore) and prefilled with 300 μl 8 M urea and 50 mM ammonium bicarbonate buffer with 5 mM of tris(2-carboxyethyl)phosphine. Samples were washed twice, denatured, and reduced at 30 °C on the Thermomixer C at 1000 RPM for 1 h followed by cysteine alkylation using 15 mM iodoacetamide for 30 min in the dark. The pH of the reaction volume was increased to around 8 by adding NH_4_OH, and isotopic labeling acetylation was performed by adding acetic anhydride-d6 (99.8% isotopic purity, Cambridge Isotopes) to 50 μM and incubating at 60 °C for 30 min. The sample was then buffer exchanged into 50 mM ammonium bicarbonate (pH 8.0) using 10K MWCO filter units. The sample was then digested with a 1:80 Glu-C-to-protein ratio for 4 h at 37 °C while shaking at 600 RPM on Thermomixer C. Glu-C digested peptides were dried down and resuspended in 50 μl of 50 mM ammonium bicarbonate (pH 8.0) and digested with trypsin at a 1:80 ratio overnight. Peptides were cleaned up with C18 tips, and the extracted peptides were lyophilized.

### LC-MS/MS

All lyophilized peptide samples were rehydrated in 2% acetonitrile, 0.1% FA and quantified by NanoDrop spectrophotometer (ThermoFisher Scientific,) prior to LC-MS/MS analysis. For acetylated samples, each was injected using a Proxeon EASY-nanoLC system coupled to a Q-Exactive Plus mass spectrometer (ThermoFisher Scientific). Peptides were separated using an analytical C18 Aurora column (75 μm × 250 mm, 1.6 μm particles; IonOpticks) at a flow rate of 300 nl/min using a 75-min gradient of 2 to 48% of solvent B (80% acetonitrile, 0.1% FA). The mass spectrometer was operated in positive data-dependent acquisition mode. MS1 spectra were measured with a resolution of 70,000, an automatic gain control (AGC) target of 1e6, a maximum injection time of 150 ms, and a mass range from 350 to 1700 mass-to-charge (*m/z*). MS2 spectra were recorded with a resolution of 35,000, AGC of 1e5, maximum IT of 110 ms, an isolation window of 1.6 *m/z*, loop count of 7, and dynamic exclusion of 5 s.

For phosphorylated samples, each was analyzed by LC-MS/MS using a Proxeon EASY-nanoLC system (Thermo Fisher) coupled to an Orbitrap Fusion Lumos mass spectrometer (ThermoFisher Scientific). Peptides were separated using an analytical C18 Aurora column at a flow rate of 300 nl/min using 75-min gradient: 1% to 6% B in 1 min, 6% to 23% B in 44 min, 23% to 34% B in 28 min, and 34% to 48% B in 2 min. The mass spectrometer was operated in positive data-dependent acquisition mode. MS1 spectra were measured in the Orbitrap in an *m/z* of 375 to 1500 with a resolution of 60,000. AGC target was set to 4 × 105 with a maximum injection time of 50 ms. The instrument was set to run in top speed mode with 2-s cycles for the survey and the MS/MS scans. After a survey scan, the most abundant precursors (with charge state between 2–7) were isolated in the quadrupole with an isolation window of 0.7 *m/z* and fragmented with higher-energy collision dissociation at 30% normalized collision energy. Fragmented precursors were detected in the orbitrap as rapid scan mode with AGC target set to 5 × 10^4^ and a maximum injection time set at 22 ms. The dynamic exclusion was set to 20 s with a 10-ppm mass tolerance around the precursor.

### Database search and analysis

All raw files were processed in the MaxQuant software (version 1.6.11.0), using the integrated Andromeda Search engine (46). MS/MS spectra were searched against the *Homo sapiens* Uniprot protein sequence database (downloaded 2020) and against a common contaminant database. Precursor mass tolerance was set to 20 ppm and 4.5 ppm for the first search where initial mass recalibration was completed for the main search, respectively. Product ions were searched with a mass tolerance of 0.5 Da, and the maximum precursor ion charge state used for searching was 7. For initial phosphorylation and acetylation data searching, carbamidomethylation of cysteines was searched as a fixed modification, whereas phosphorylation, lysine acetylation, and oxidation were set as variable modifications. “Enzyme” was set to trypsin and a maximum of two missed cleavages were allowed. For assessment of acetylation stoichiometry, both unlabeled and “heavy” acetyl-lysine were set as variable modifications. The “heavy” acetyl-lysine must be added to MaxQuant by the user following MaxQuant’s instructions with the “Acetyl_heavy” modification from the Unimod database (www.unimod.org/). “Enzyme” was set to Glu-C and trypsin in a specific mode and a maximum of four missed cleavages were allowed for searching and the minimum peptide length was set 5. The target-decoy-based false discovery rate filter for spectrum and protein identification was set to 1%.

### EMSA of premodified TFAM

Two hundred nanograms of pUC19 DNA was compacted with unmodified TFAM, acetyl-TFAM, or phosphor-TFAM at a 50 bp DNA:TFAM ratio at 37 °C for 30 min in the previously described incubation buffer. A “hPKAc control” sample was included in which pUC19 DNA was incubated with hPKAc to control for the small amount of hPKAc remaining in the phosphor-TFAM stock after buffer exchange. A 1% agarose EMSA gel was run as described above and stained with SYBR Gold (ThermoFisher Scientific) for 1 h to visualize the DNA.

### Tailed-template preparation and promoter-independent transcription assays

DNA oligonucleotides for tailed transcription templates (Fig. S7) were gel-purified of by electrophoresis through an 8% polyacrylamide gel (19:1 acrylamide:bisacrylamide) containing 8 M urea and TBE buffer (1.25 mM Na_2_EDTA and 44.5 mM Tris-borate, pH 8.3). Gel pieces containing the oligos were excised and incubated overnight in low salt oligo purification buffer (10 mM Tris–Cl pH 7.5, 1 mM EDTA, 100 mM NaCl). The extracted nucleic acids were then purified on DEAE Sepharose resin (Bio-Rad), eluted with high salt oligo purification buffer (10 mM Tris–Cl pH 7.5, 1 mM EDTA, 2 M NaCl), and ethanol-precipitated. Gel-purified oligonucleotides were then annealed in a thermocycler by heating to 95 °C for 5 min followed by gradual cooling to 25 °C at 1 °C per minute. DNA templates were compacted with TFAM for 30 min at 37 °C in a transcription buffer (25 mM Tris–HCl pH 8.0, 70 mM NaCl, 10 mM MgCl_2_, 100 μg/ml bovine serum albumin, 1 mM DTT) at the DNA bp:TFAM ratios of 50 (∼94 nM), 25 (∼189 nM), and 12 (∼379 nM). Compacted DNA samples were then treated with 150 nM POLRMT and [α-^32^P]-ATP (PerkinElmer) for 20 min to initiate labeled RNA synthesis before introducing an NTP mix (150 μM GTP, 150 μM CTP, 10 μM UTP, and 400 μM ATP) into the reaction (all NTPs were purchased as HPLC-purified from Cytiva). The reaction was quenched using a 2X stop-buffer (90 mM Tris–borate, 8 M urea, 50 mM EDTA, 0.02% xylene cyanol, and 0.02% bromophenol blue) after 30 min, boiled for 8 min, and spun down to pellet protein before loading onto an 8 M Urea/6% polyacrylamide gel. The resulting gel was imaged as previously mentioned and quantified using ImageQuant software.

### EMSA analysis of poly-T ssDNA

A 30-nucleotide thymine-only ssDNA oligo (Eton Biosciences) was 5′ end-labeled with [γ -^32^P]- ATP using T4 polynucleotide kinase (New England Biolabs) according to manufacturer instructions and heat inactivated. The ssDNA oligo was then annealed to a poly-A oligo (Eton Biosciences) using the annealing protocol mentioned above. Forty nanomolar poly-T ssDNA and poly-T dsDNA were then incubated with TFAM at 50 bp:TFAM, 25 bp:TFAM, and 12 DNA bp:TFAM at the same concentrations as the tailed-transcription assay within the transcription buffer mentioned above at 37 °C for 30 min before loading onto a ∼6% polyacrylamide gel (50 mM Tris–acetate pH 8.0, 2.5 mM EDTA) with 5% glycerol. The gel was run at 70 V at room temperature and imaged as previously mentioned.

### Promoter-dependent transcription assays

Transcription was carried out using the fully dsDNA template containing mitochondrial LSP promoter (-42 to +21, numbering based on human mitochondrial genome convention), prepared by annealing complementary oligos after gel-purification as described above (Fig. S6). One hundred fifty nanomolar template, 150 nM POLRMT, 150 nM TFB2M, 150 nM TFAM, and [α-^32^P]-ATP (PerkinElmer) were incubated in the transcription buffer above to initiate labeled RNA synthesis and mixed with 150 μM GTP, 150 μM dCTP (to control the length of the elongated RNA product), 10 μM UTP, and 400 μM ATP (NTP mix recipe from Ramachandran *et al*. (11). The reaction was allowed to proceed for 15 min at 37 °C before quenching with the above 2X stop buffer and preparation for gel electrophoresis as previously described. Samples were run out on an 8 M urea/15% polyacrylamide gel and imaged as previously described.

## Data availability

Raw data are available on the MassIVE database at the following link: doi:10.25345/C58W0Z.

## Supporting information

This article contains supporting information (Figs. S1–S7 and Table S1).

## Conflict of interest

The authors declare that they have no conflicts of interest with the contents of this article.

## Supporting information

Supplemental Figures S1–S8 and Table S1

Reardon_TFAM_SuppTable_AceticAnhydrideResultsandWorkup

Reardon_TFAM_SuppTable_All PTM
