# Supplementary material for: Phosphorylation and acetylation of mitochondrial transcription factor A promote transcription processivity without compromising initiation or DNA compaction
Source: J Biol Chem. 2022 Mar 10;298(4):101815. doi: 10.1016/j.jbc.2022.101815 (PMC9006650; doi:10.1016/j.jbc.2022.101815)
Supplement: Supplemental Figures S1–S8 and Table S1 [file mmc1.pdf]

Supporting Information for:

Phosphorylation and acetylation of mitochondrial transcription factor A promote transcription processivity without compromising initiation or DNA compaction

Sean D. Reardon[1] and Tatiana V. Mishanina*,[1]

[1]Department of Chemistry and Biochemistry University of California, San Diego, 9500 Gilman Dr, La Jolla, CA 92093

List of contents:

Fig. S1: ImageQuant gel analysis of TFAM phosphorylation by hPKA

Fig. S2: Purification SDS-PAGE gel of the proteins used in this study and TFAM phosphorylation analysis at varying DNA compaction levels

Fig. S3: Peptide mass spectra of acetylated and phosphorylated TFAM residues

Fig. S4: TFAM acetylation site assignment by intensity without isotopic labelling

Fig. S5: Example MS1 spectra of light/heavy acetyl-peptide

Fig. S6: Analysis of TFAM acetylation stoichiometry

Fig. S7: Transcription templates

Fig. S8: Poly-T ssDNA EMSA

Table S1: Protein purification buffers

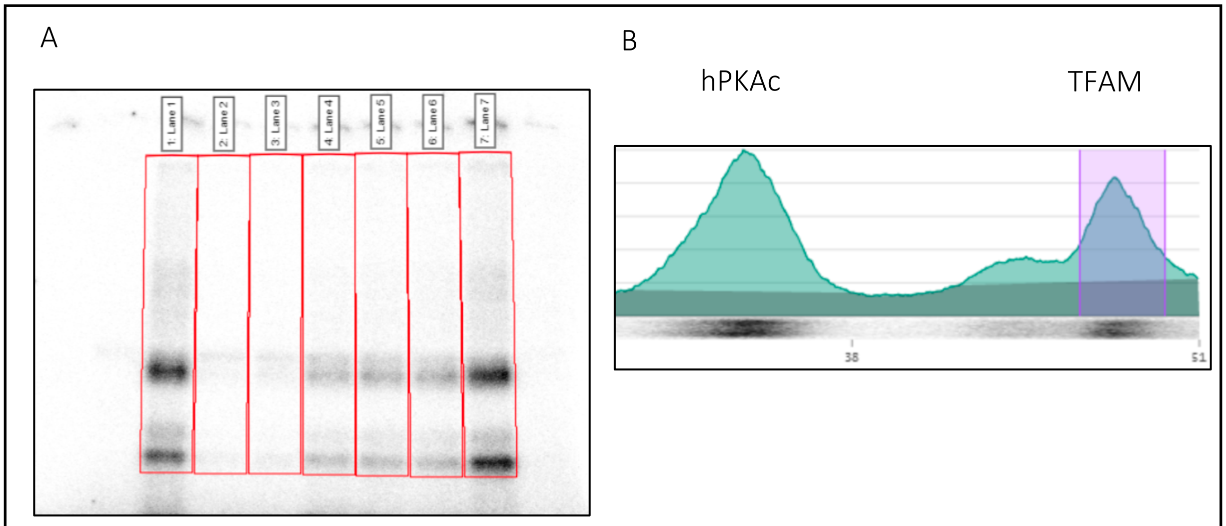

C

$$Phosphorylated\ TFAM = \frac{Phosphor\ Peak}{Coomassie\ Peak}$$

Figure S1. Example image analysis of phosphorylated TFAM. A: ImageQuant software allows assigning lanes in a phosphor (shown) and Coomassie-stained gel image before quantifying peaks of a band. The gel shown as an example is the same as in Fig. S2C below. B: Representative image of assigning band and analyzing peak values in ImageQuant. C: The equation used to normalize the phosphor signal to the protein content of a band within a given TFAM phosphorylation experiment.

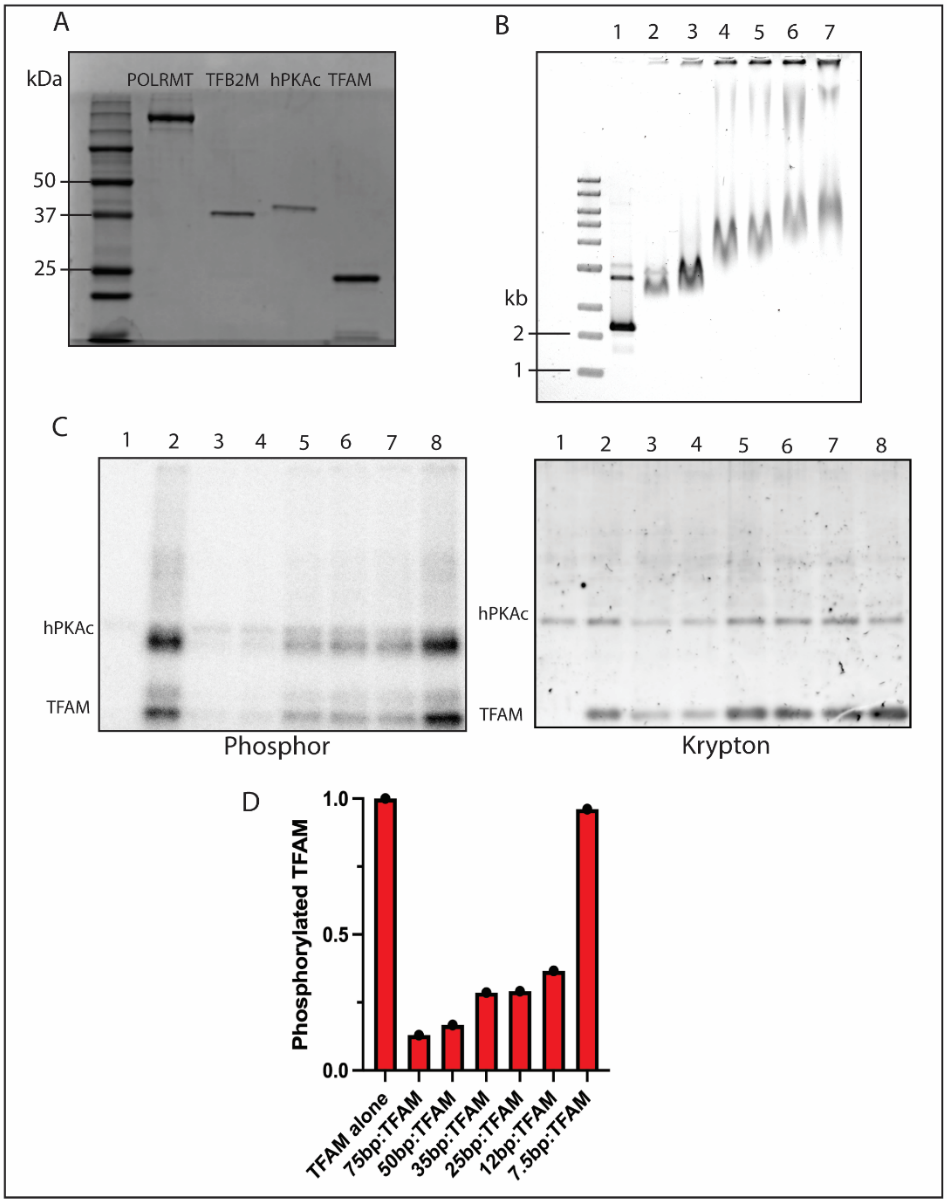

Figure S2. TFAM phosphorylation analysis at varying DNA compaction levels. A: Purified mitochondrial proteins. B: EMSA gel analysis of compacted pUC19 DNA with TFAM at varying DNA:protein ratios. Lanes 1: pUC19 DNA only, 2: 75bp:TFAM, 3: 50bp:TFAM, 4: 35bp:TFAM, 5: 25bp:TFAM, 6: 12bp:TFAM, 7: 7.5bp:TFAM. C: *In vitro* phosphorylation of TFAM by hPKAc across a range of compaction levels analyzed by phosphor imaging and Krypton staining. Lanes 1: hPKAc alone, 2: TFAM, 3: 75bp:TFAM, 4: 50bp:TFAM, 5: 35bp:TFAM, 6: 25bp:TFAM, 7: 12bp:TFAM, 8: 7.5bp:TFAM. D: Phosphorylated TFAM from C was quantified using ImageQuant.

Figure S3. Peptide mass spectra of acetylated and phosphorylated TFAM residues.

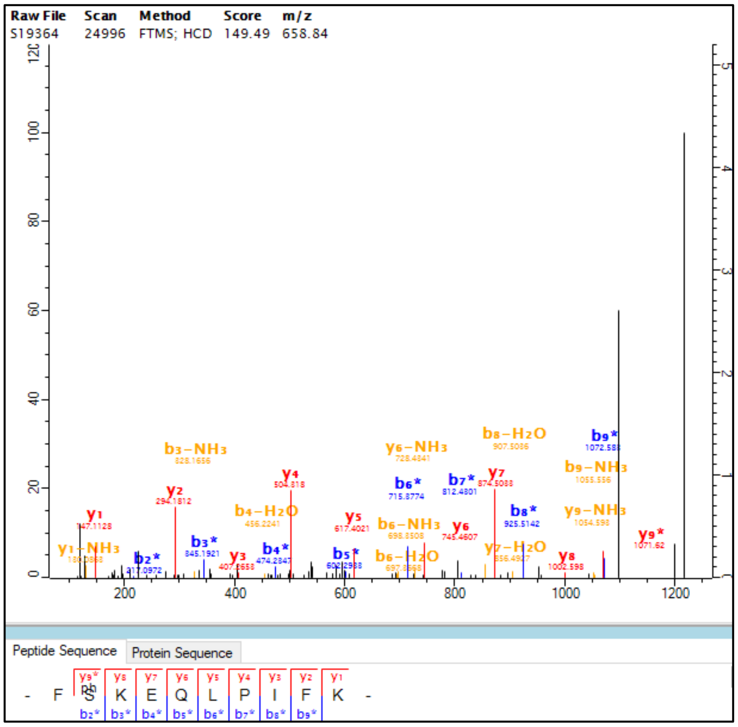

Peptide containing phospho-S61

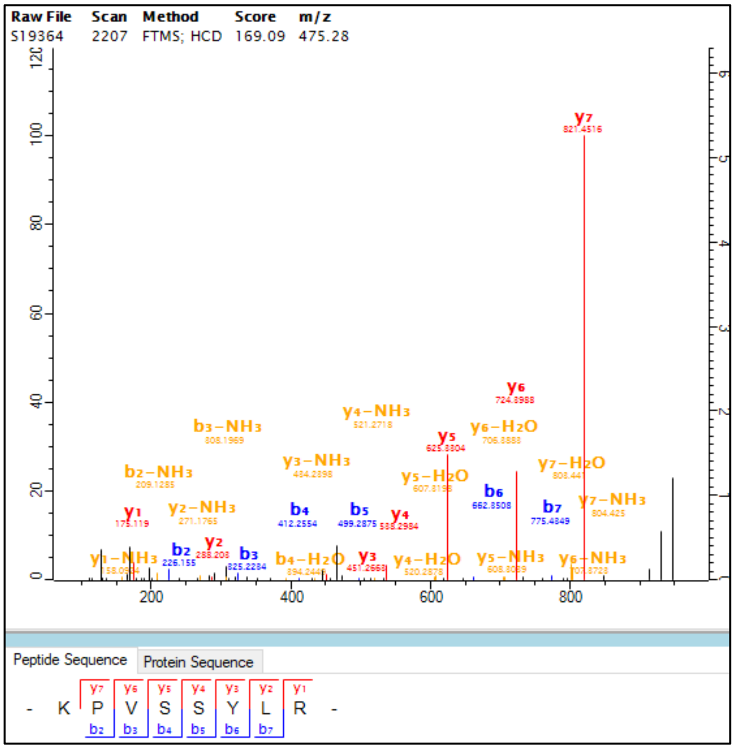

Peptide for residues 52-59 (containing S55 and S56) was <u>unmodified</u> in the phospho-TFAM sample

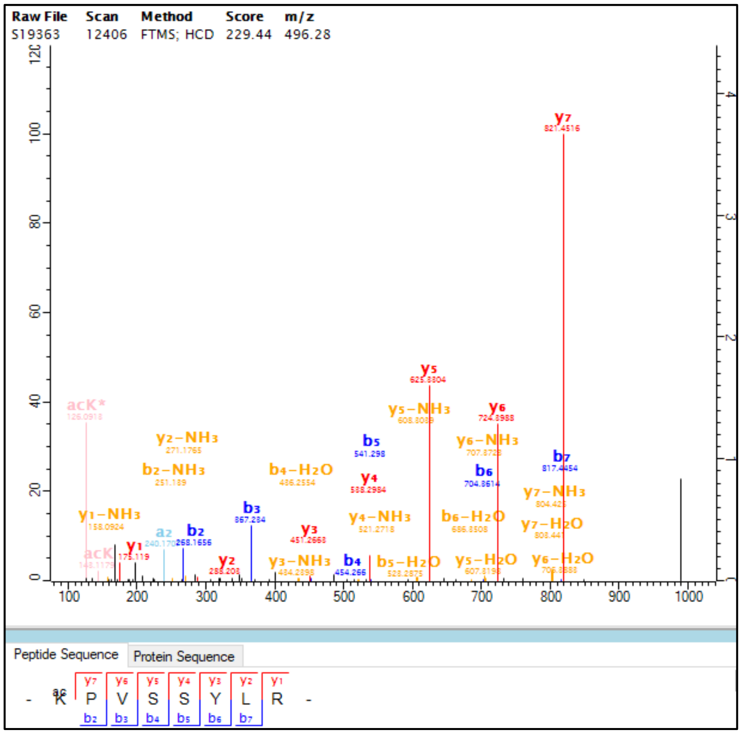

Peptide containing acetyl-K52

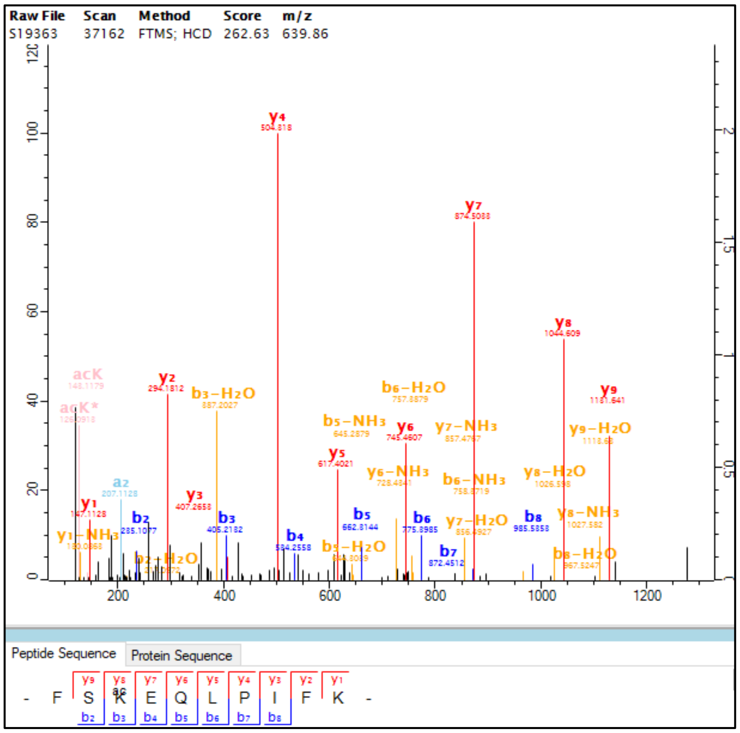

Peptide containing acetyl-K62

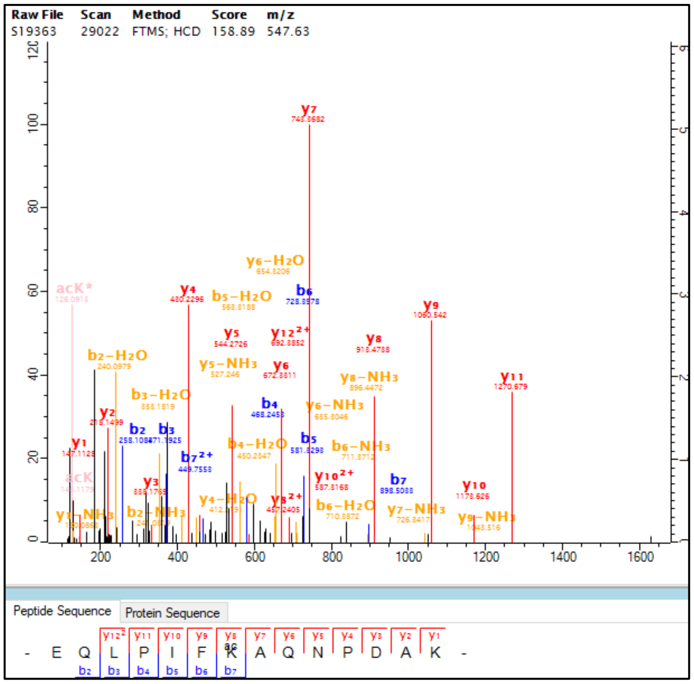

Peptide containing acetyl-K69

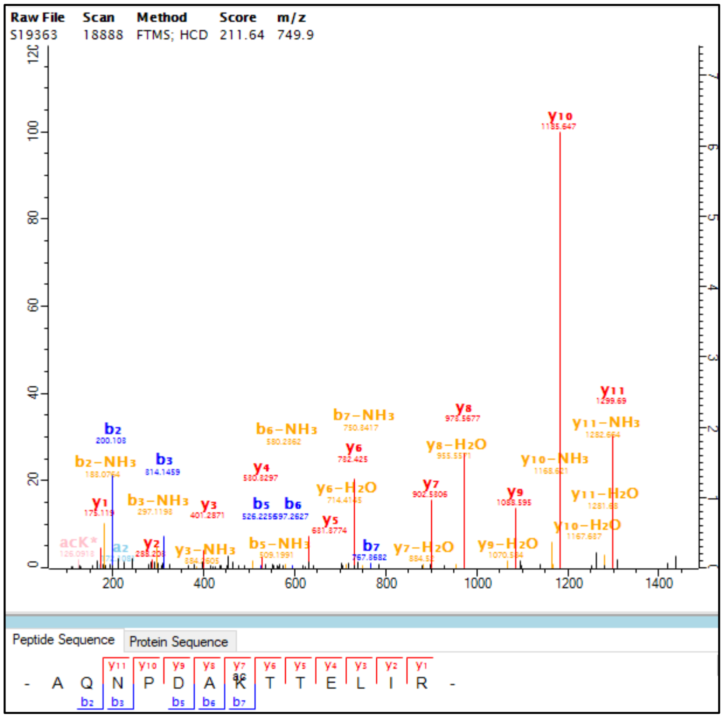

Peptide containing acetyl-K76

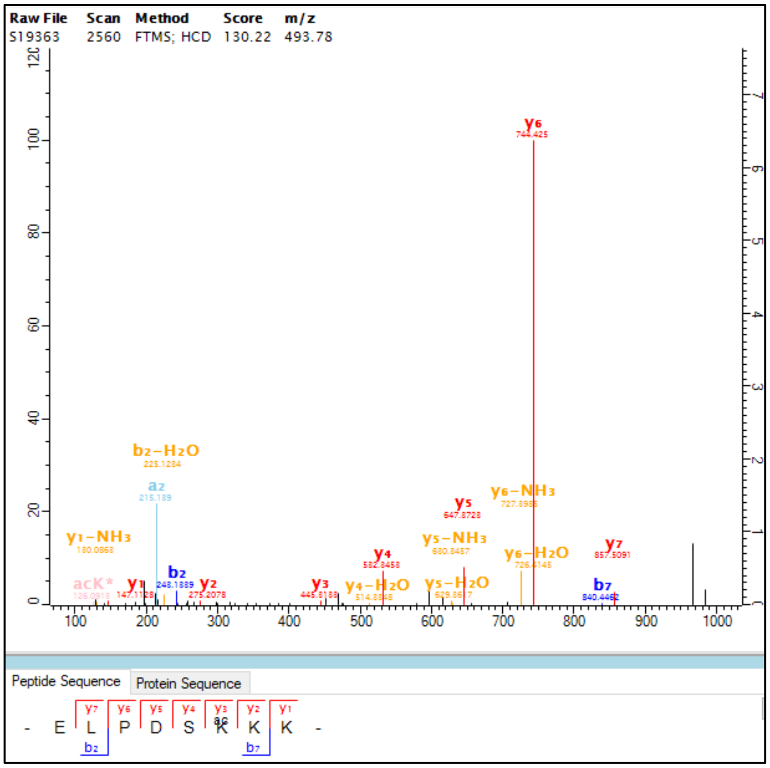

Peptide containing acetyl-K95

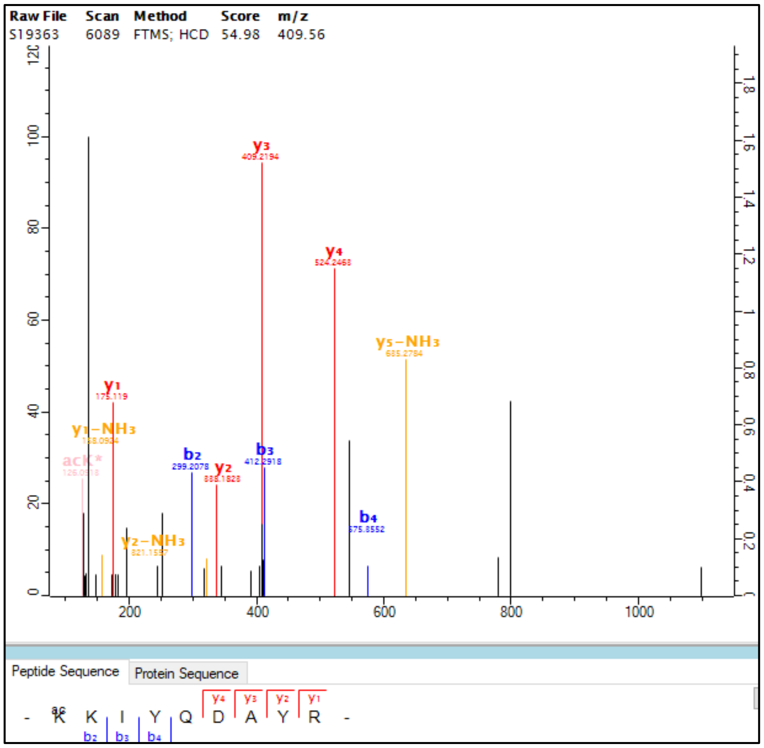

Peptide containing acetyl-K96

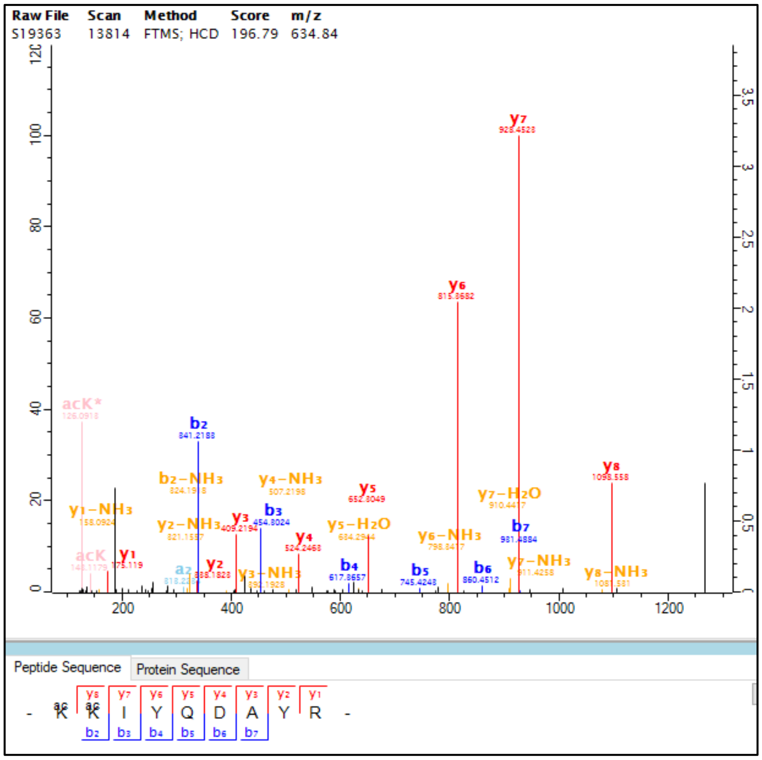

Peptide containing acetyl-K96 an acetyl-K97

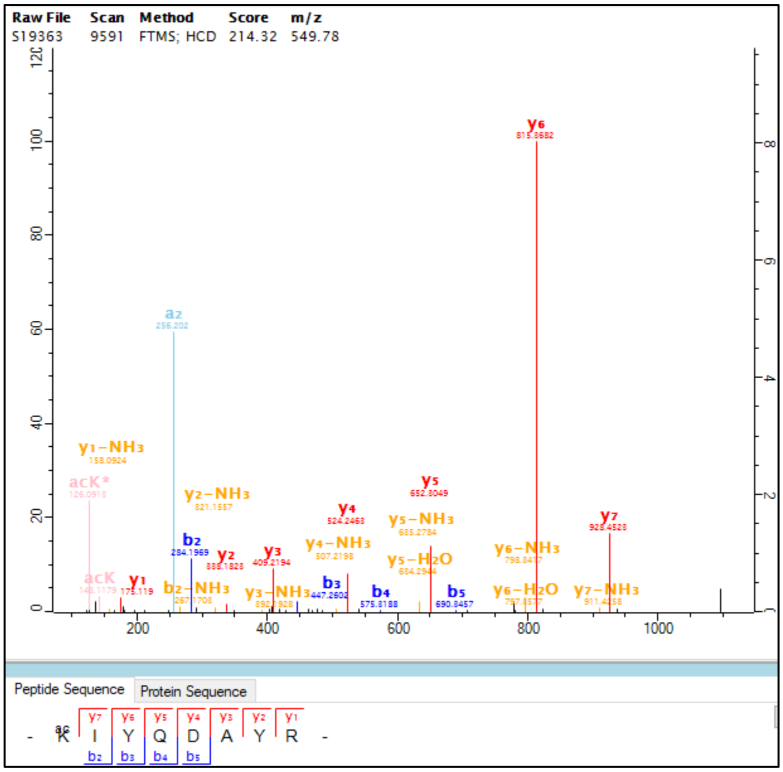

Peptide containing acetyl-K97

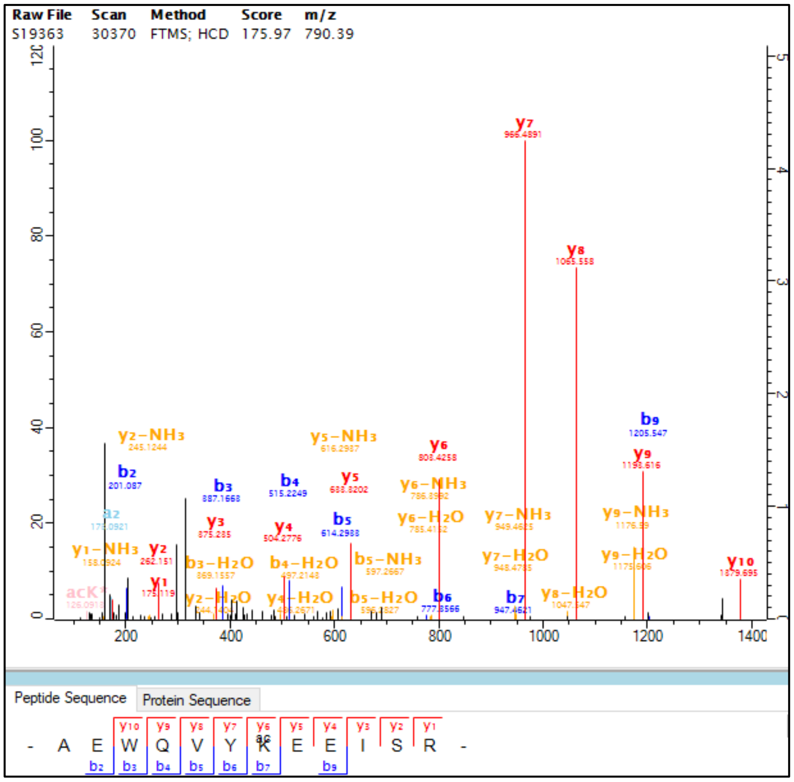

Peptide containing acetyl-K111

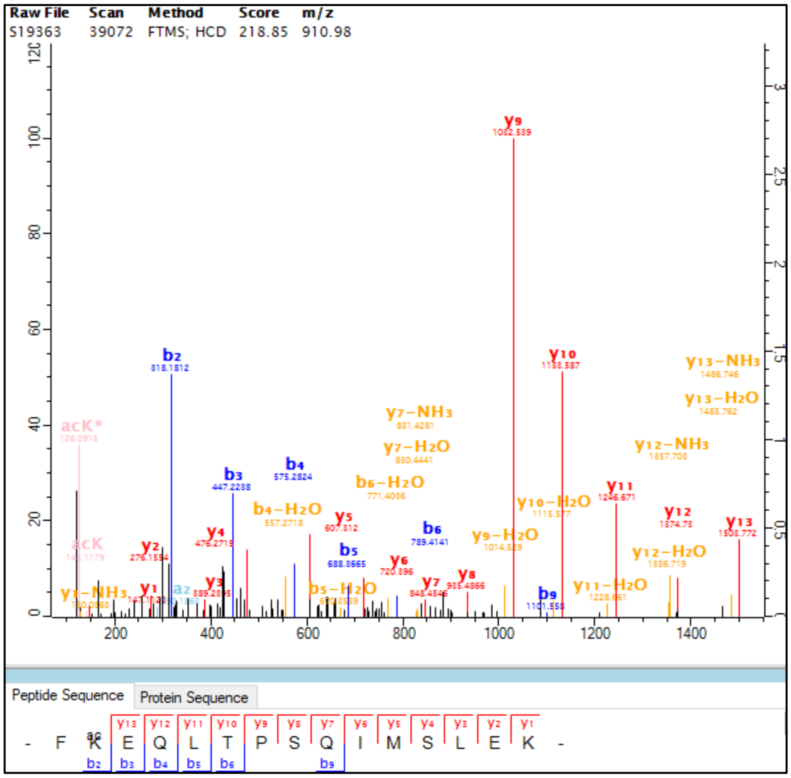

Peptide containing acetyl-K118

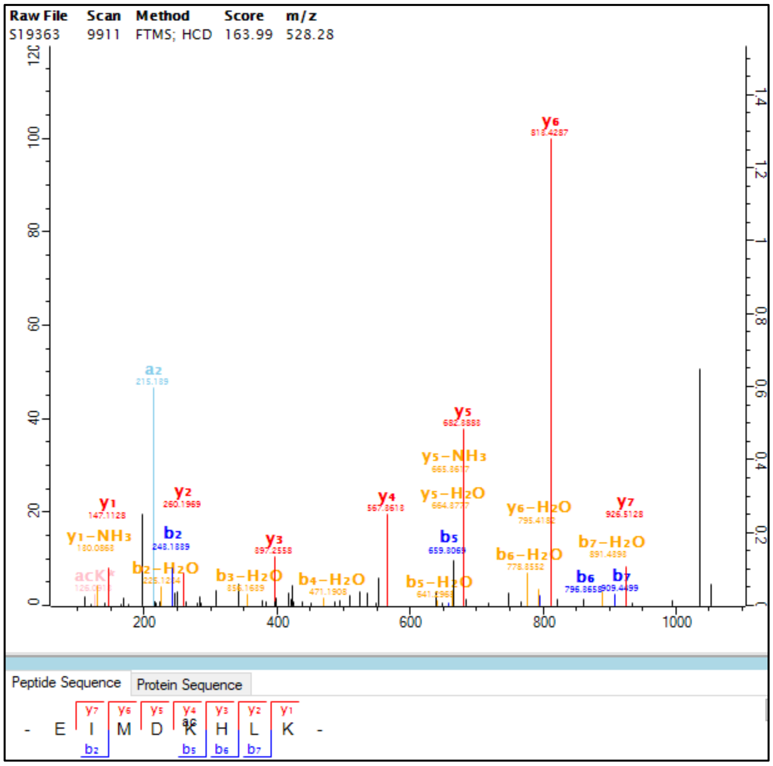

Peptide containing acetyl-K136

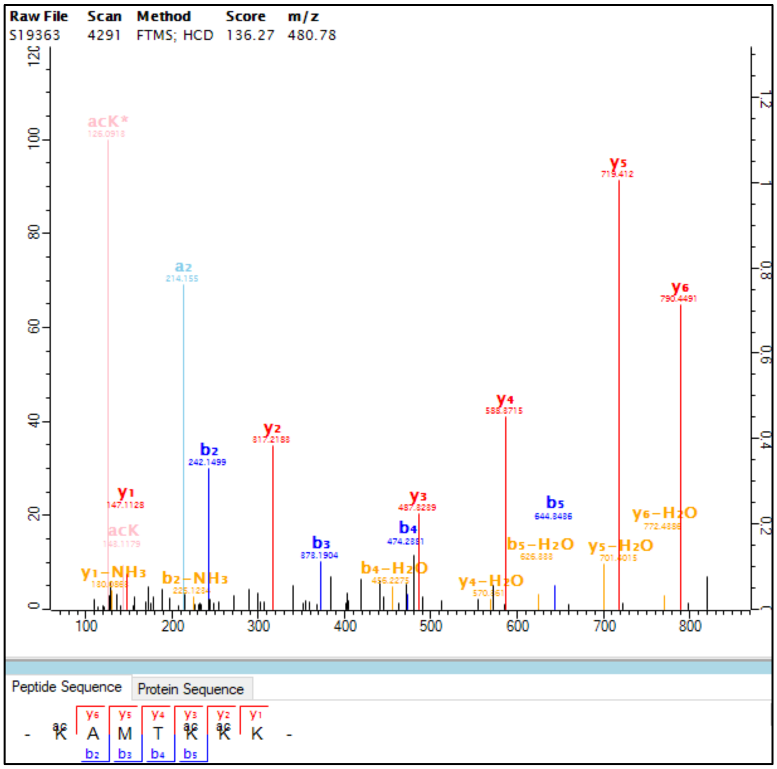

Peptide containing acetyl-K141, acetyl-K145, and acetyl-K146

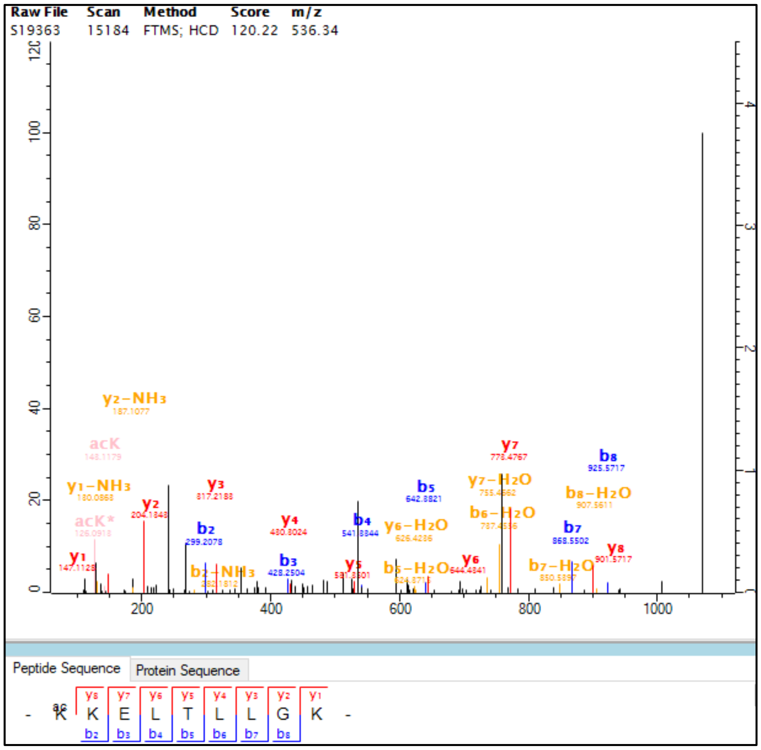

Peptide containing acetyl-K146

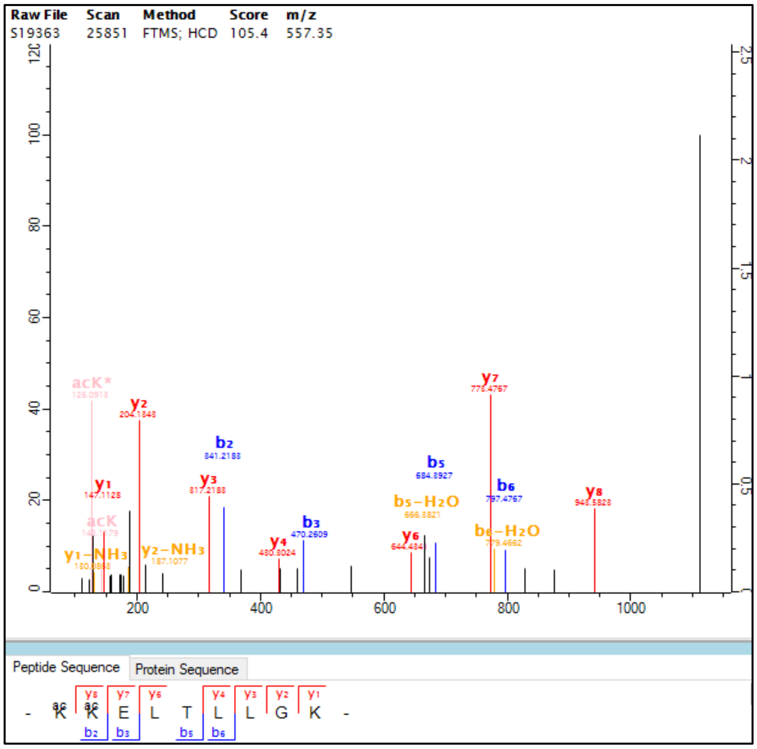

Peptide containing acetyl-K146 and acetyl-K147

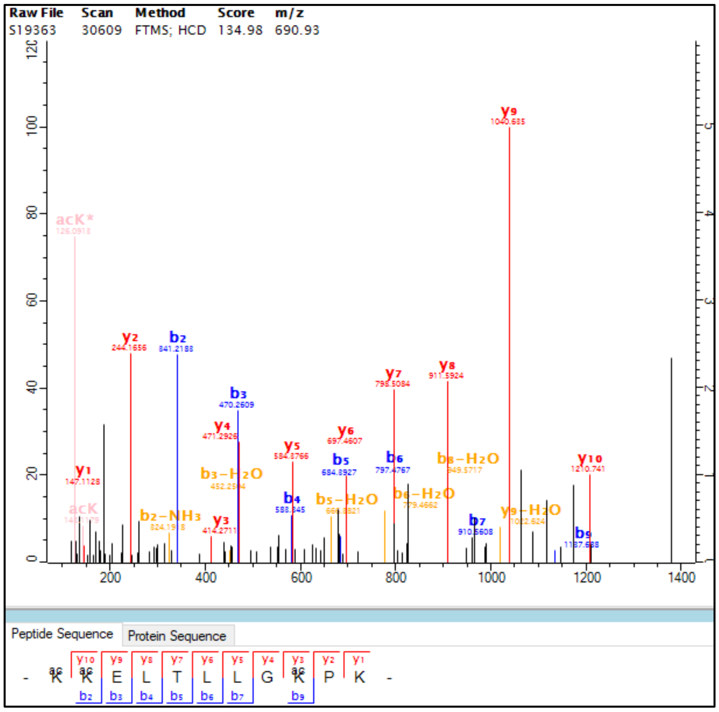

Peptide containing acetyl-K146, acetyl-K147, and acetyl-K154

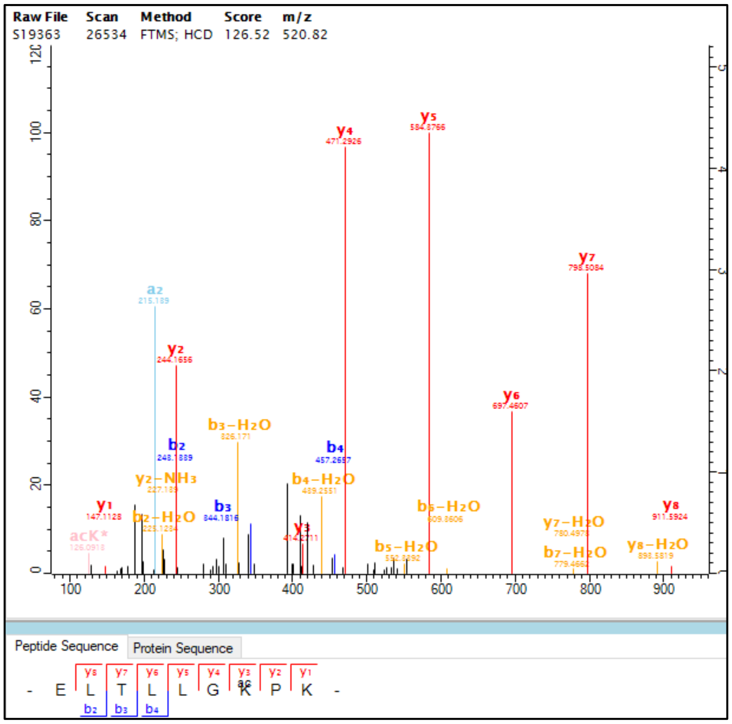

Peptide containing acetyl-K154

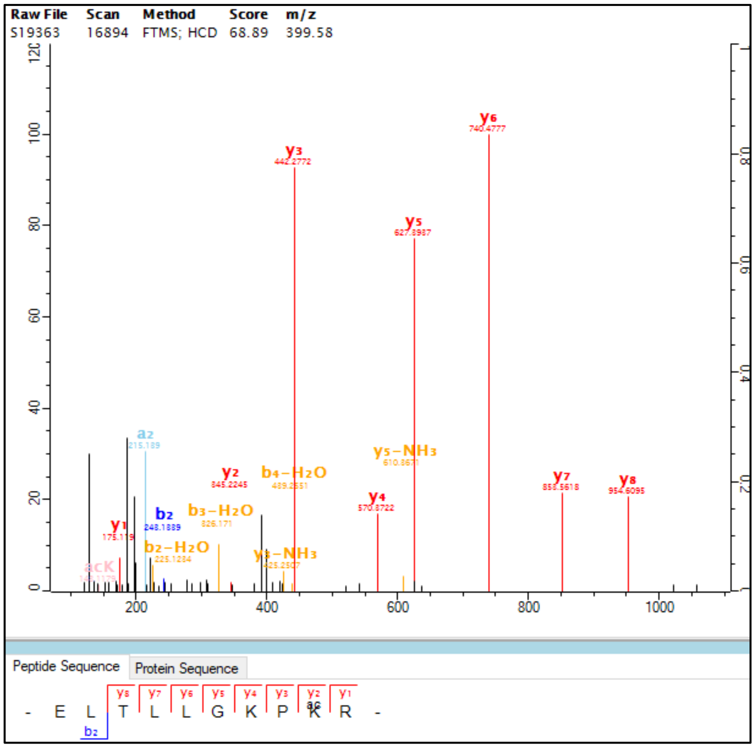

Peptide containing acetyl-K156

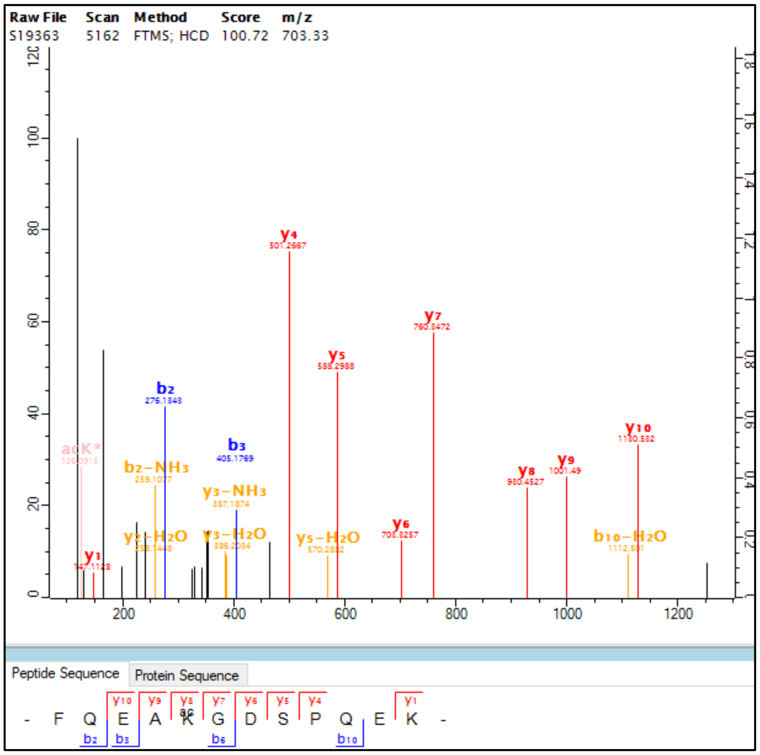

Peptide containing acetyl-K174

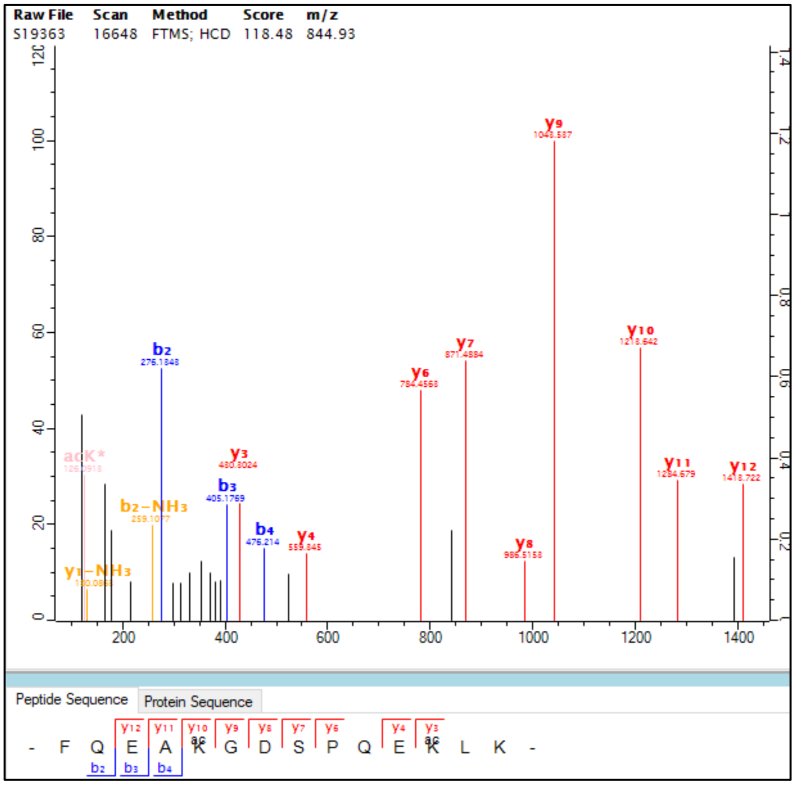

Peptide containing acetyl-K174 and acetyl-K181

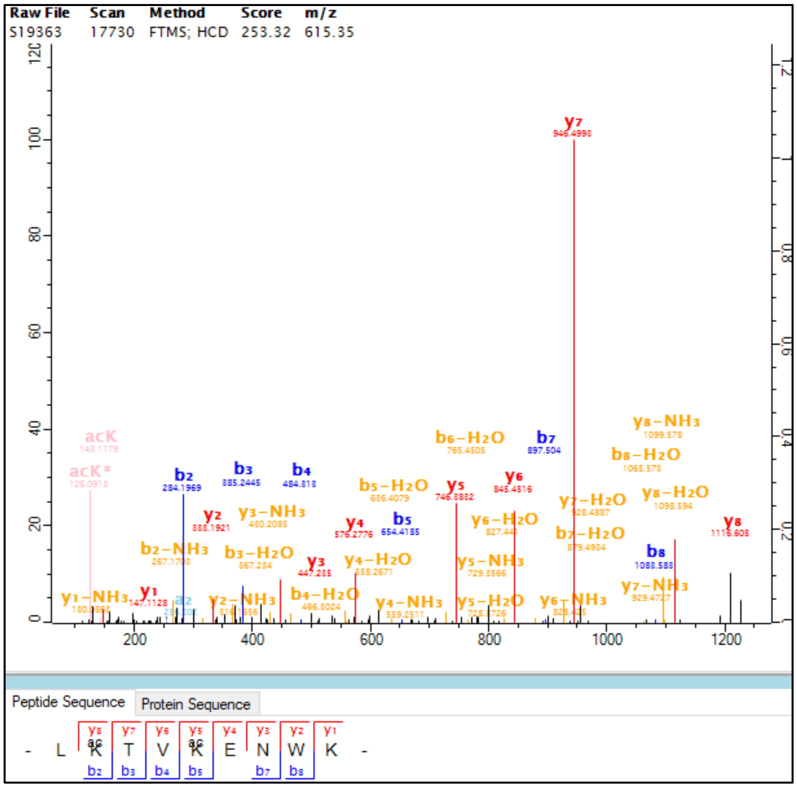

Peptide containing acetyl-K183 and acetyl-K186

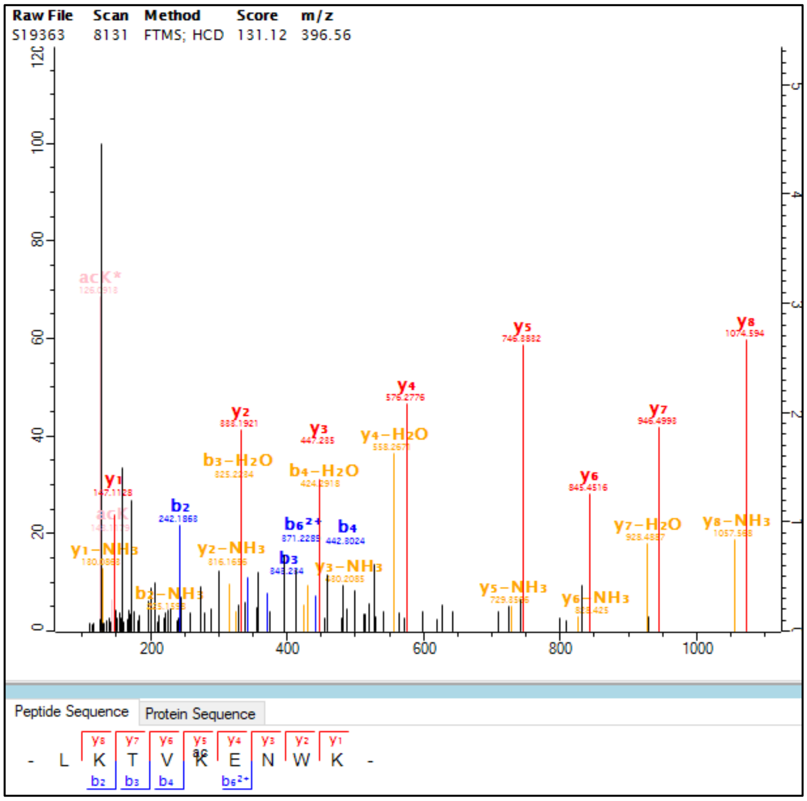

Peptide containing acetyl-K186

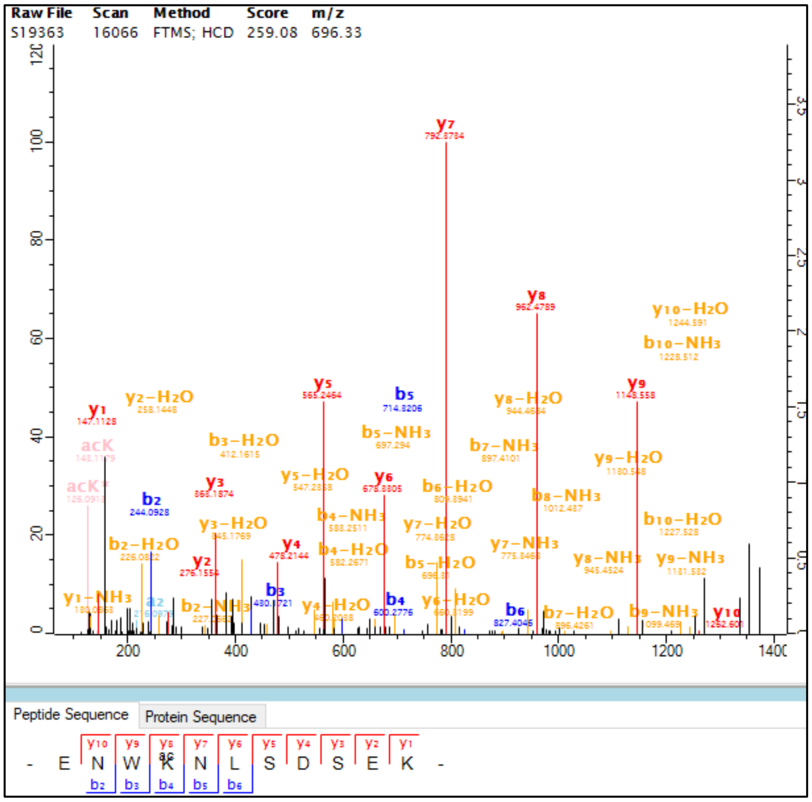

Peptide containing acetyl-190

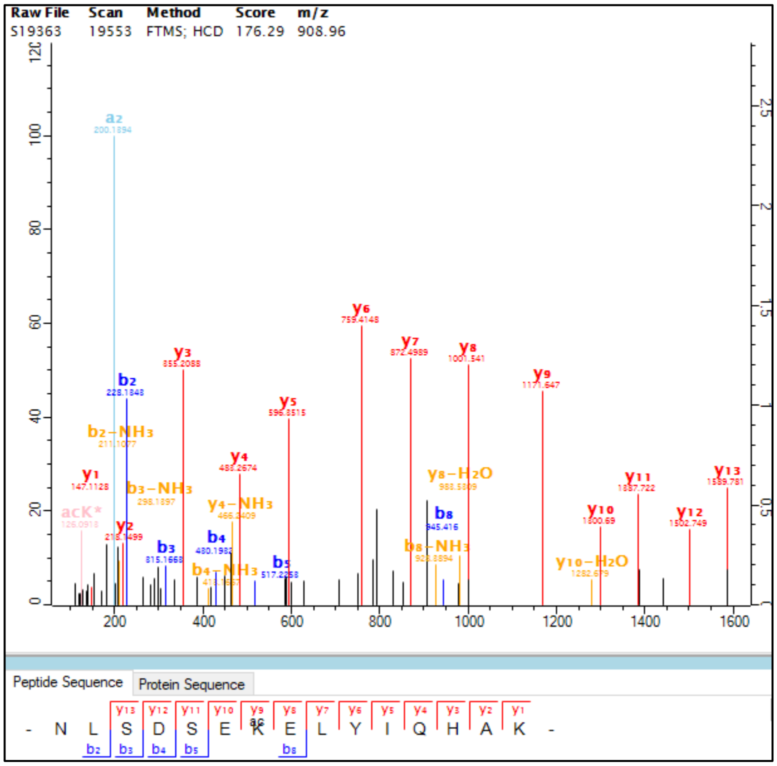

Peptide containing acetyl-K197

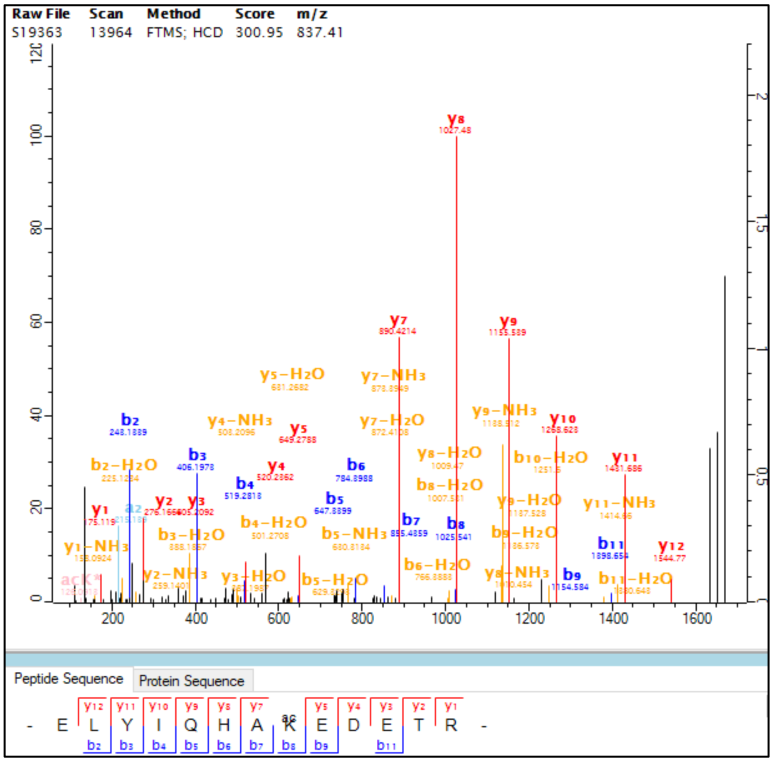

Peptide containing acetyl-K205

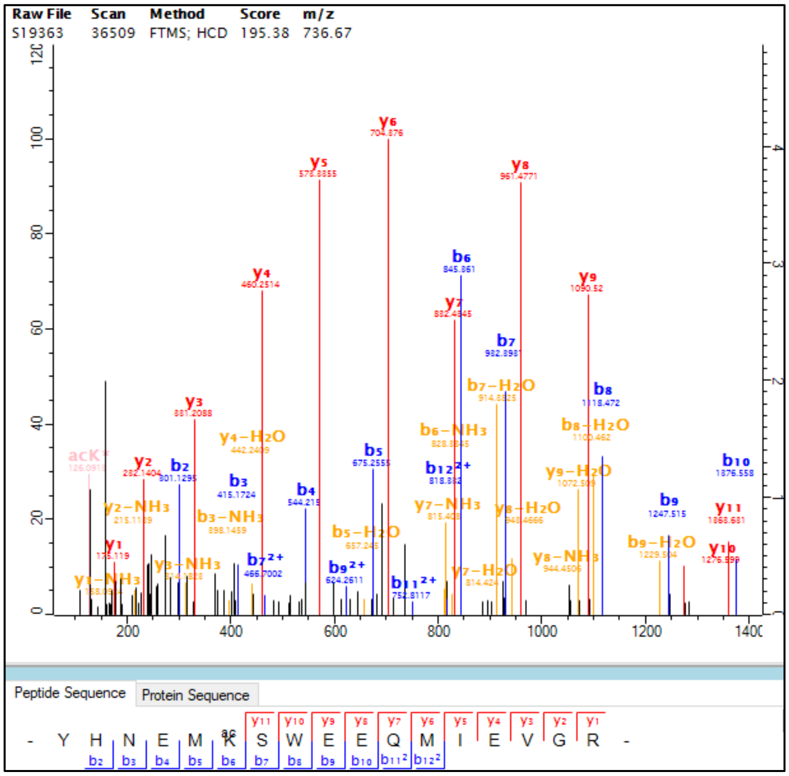

Peptide containing acetyl-K216

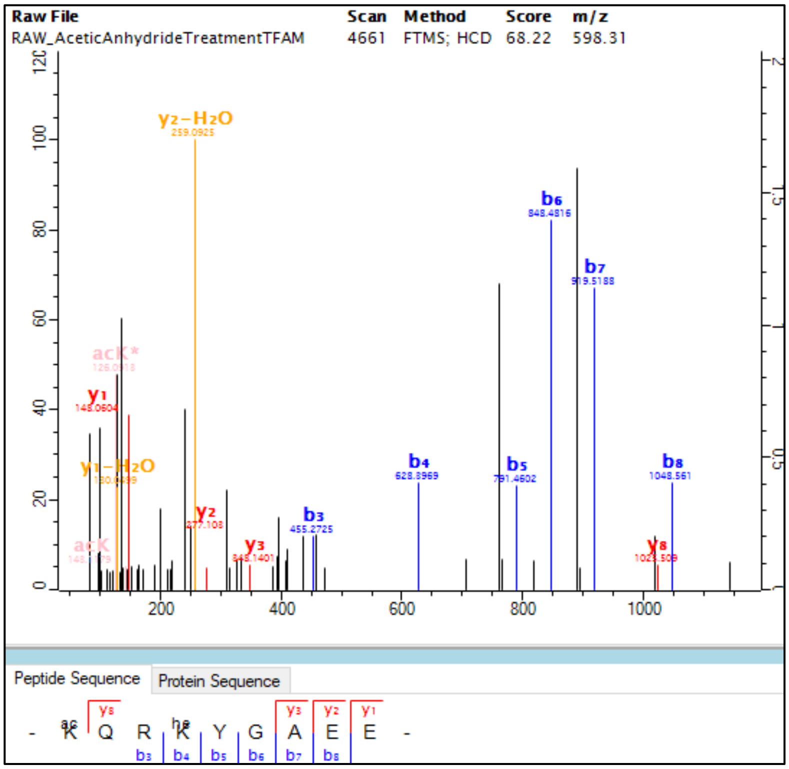

Peptide containing acetyl-K237 and acetyl-K240 from the acetic anhydride treated TFAM sample

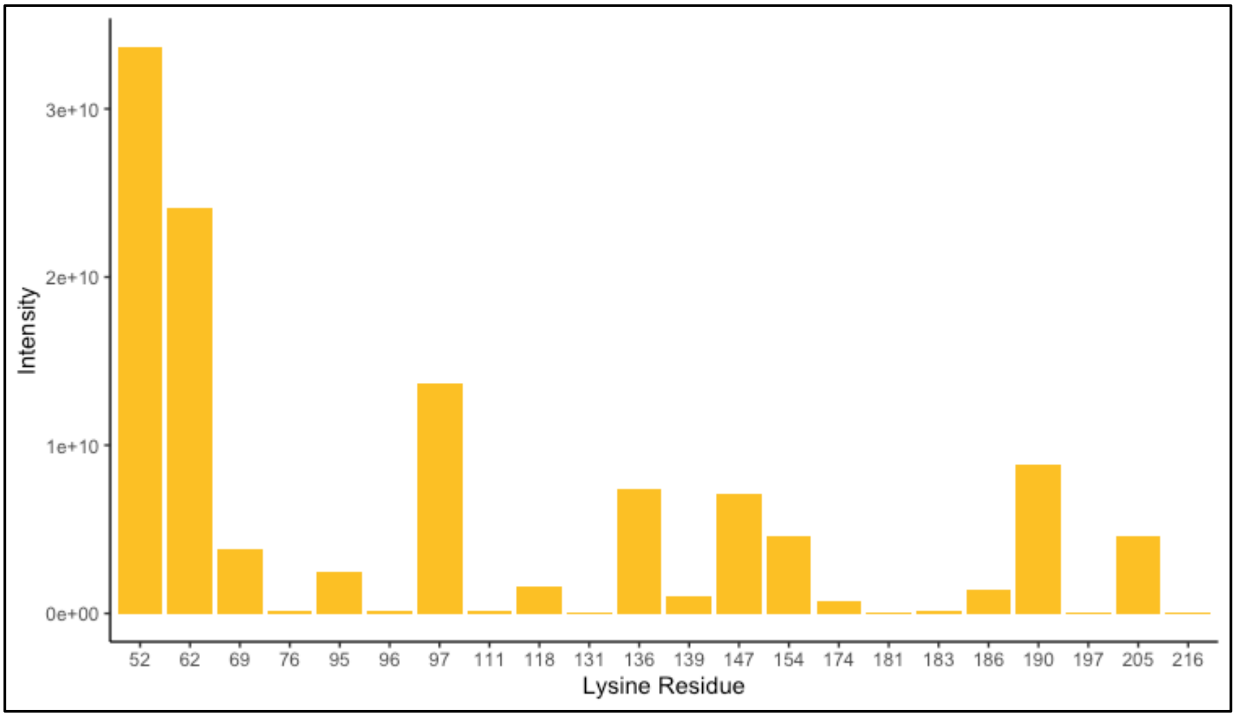

Figure S4. The intensity of peptides assigned to each acetyl-lysine residue in MaxQuant after LC-MS/MS with trypsin digestion. Acetylated lysines were first identified without isotopic labelling using only a tryptic digest, and MaxQuant, allowing only 2 missed cleavages. Intensities shown for each residue are those assigned to the residue within the “Acetyl (K) Sites” table of MaxQuant’s output.

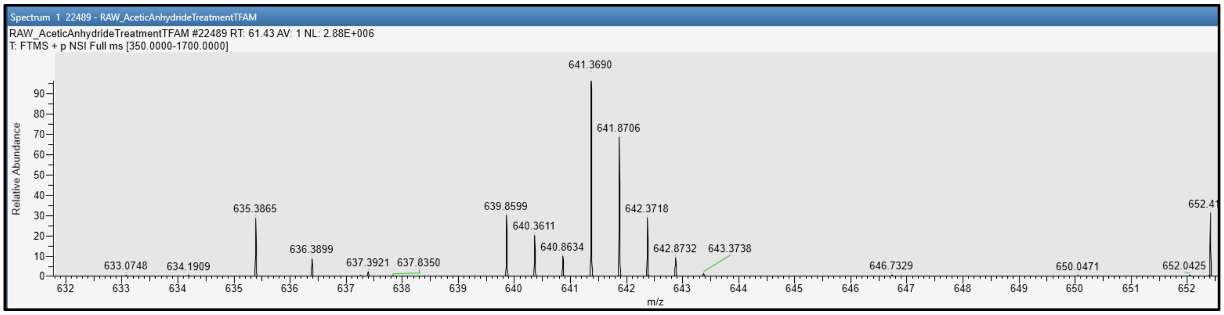

Figure S5. A screenshot from the FreeStyle Program (ThermoFisher Scientific) representing MS1 spectra of the peptide containing acetyl K62. The left peaks represent the light acetyl modification and directly to the right is the heavy version of the peptide.

$$
\begin{aligned}
&\mathit{Stock\ acetylation\ on\ a\ given\ residue} \\
&\quad = (\mathit{Light\ Intensity})/(\mathit{Heavy\ Intensity} \\
&\quad + \mathit{Light\ Intensity})
\end{aligned}
$$

Figure S6. Analysis formula for comparing light and heavy acetyl groups at each residue using the intensities given in the acetyl-K and Heavy acetyl-K tables from the MaxQuant search.

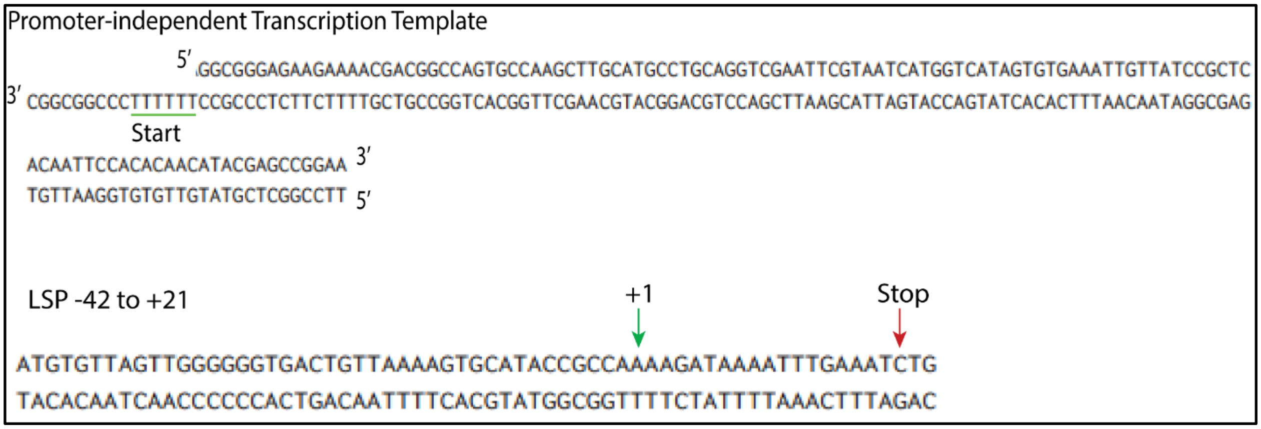

Figure S7. Transcription templates for the promoter-independent and promoter dependent (Light-Stand Promoter, or LSP) transcription assays in Fig. 5 of the main text. +1 indicates transcription start site, and “Stop” refers to the position at which dCTP is incorporated into RNA.

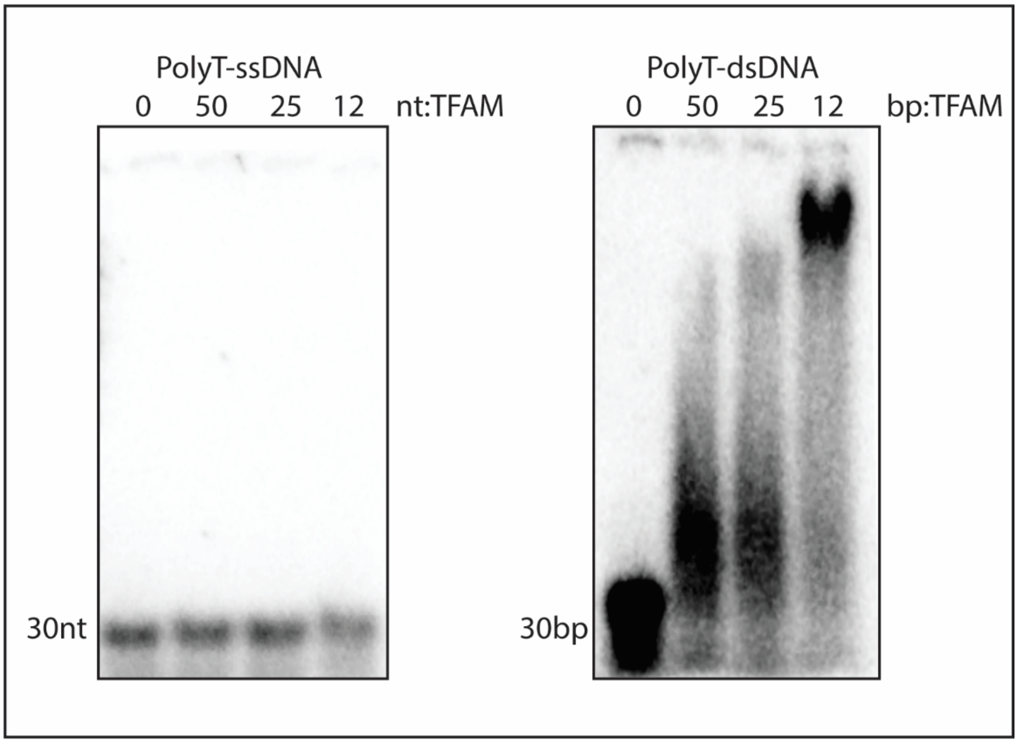

Figure S8. EMSA gel analysis of TFAM incubated with poly-T ssDNA (left) at various concentrations vs. poly-T dsDNA (right) shows that TFAM has very low affinity for unstructured ssDNA and thus does not compete with POLRMT transcription initiation at the single-stranded overhang in tailed-transcription experiments (Fig. 5 in the main text).

Table S1. Components for buffers used in protein purification

Protein	Buffer	Components
TFAM	Ni Buffer A	Tris-HCl pH 7.5, 250mM NaCl, 10mM imidazole, 10% glycerol, 1X protease inhibitor cocktail (1XPI, Roche), 1mM BME
TFAM	Ni Buffer B	Tris-HCl pH 7.5, 250mM NaCl, 0.5M imidazole, 10% glycerol, 1XPI, 1mM BME
TFAM	Heparin Buffer A	Tris-HCl pH 7.5, 250mM NaCl, 10% glycerol, 1XPI, 1mM BME
TFAM	Heparin Buffer B	Tris-HCl pH 7.5, 1M NaCl, 10% glycerol, 1XPI, 1mM BME
TFAM	Storage	Tris-HCl pH 7.5, 100mM NaCl, 40% glycerol, 1mM DTT
TFB2M	Ni Buffer A	20mM HEPES-KOH pH 8.0, 0.3M KCl, 5% glycerol, 20mM imidazole, 1XPI, 1mM BME
TFB2M	Ni Buffer B	20mM HEPES-KOH pH 8.0, 1MKCl, 5% glycerol, 500mM imidazole, 1XPI, 1mM BME
TFB2M	Heparin Buffer A	20mM HEPES-KOH pH 8.0, 200mM KCl, 5% glycerol, 1mM EDTA, 1XPI, 1mM BME
TFB2M	Heparin Buffer B	20mM HEPES-KOH pH 8.0, 1.5M KCl, 5% glycerol, 1mM EDTA, 1XPI, 1mM DTT
TFB2M	Storage	20mM HEPES-KOH pH 8.0, 200mM KCl, 25% glycerol, 1mM DTT
POLRMT	Ni Buffer A	40mM Tris-HCl pH 7.9, 300mM NaCl, 20mM imidazole, 15% glycerol, 0.1% Tween-20, 0.3mM EDTA, 1XPI, 1mM DTT
POLRMT	Ni Buffer B	40mM Tris-HCl pH 7.9, 300mM NaCl, 0.5M imidazole, 15% glycerol, 0.1% Tween-20, 0.3mM EDTA, 1XPI, 1mM DTT
POLRMT	Heparin Buffer A	40mM Tris-HCl pH 7.9, 150mM NaCl, 15% glycerol, 0.1% Tween-20, 1mM EDTA, 1XPI, 1mM DTT
POLRMT	Heparin Buffer B	40mM Tris-HCl pH 7.9, 1M NaCl, 15% glycerol, 0.1% Tween-20, 1mM EDTA, 1XPI, 1mM DTT
POLRMT	Storage	40mM Tris-HCl pH 7.9, 150mM NaCl, 50% glycerol, 0.1% Tween-20, 1mM EDTA,1mM DTT

hPKAc	Ni Buffer A	20mM Tris-HCl pH 8.0, 300 mM NaCl, 1XPI, 2 mM BME
hPKAc	Ni Buffer B	20mM Tris-HCl pH 8.0, 300 mM NaCl, 150mM imidazole, 1XPI, 2 mM BME
hPKAc	Storage	20mM MES pH 6.5, 200 mM NaCl, 60% glycerol, 2 mM DTT
